# Targeting T-Cells for Cancer Treatment: Current Clinical Strategies and Challenges

**DOI:** 10.3390/biomedicines14030654

**Published:** 2026-03-13

**Authors:** Anand Rotte, Mentor Sopjani, Madhuri Bhandaru

**Affiliations:** 1 Regulatory Affairs, Arcellx Inc., Redwood City, CA 94065, USA; 2 Faculty of Medicine, University of Prishtina, 10000 Prishtina, Kosovo; mentor.sopjani@uni-pr.edu; 3 Drug Safety and Pharmacovigilance, Amgen Inc., Thousand Oaks, CA 91320, USA

**Keywords:** immunotherapy, effector T-cells, BiTEs, PD-1, CAR T-cells and TIL therapy

## Abstract

Modulation of immune response to target tumor cells has been shown to be a successful strategy for cancer treatment. Over the past years immunotherapy has been integrated into cancer treatment and PD-1 blockers have become the backbone of treatment regimens for multiple cancer types. Several classes of immunotherapies, such as immune checkpoint blockers, bispecific antibodies, chimeric antigen receptor (CAR) T-cells, and tumor-infiltrating lymphocytes (TILs), were approved by the US FDA in the last decade and many more are in clinical trials. Research on redirecting effector T-cells to treat cancer has been aimed at addressing the limited responses in solid tumors, emergence of resistance, treatment-limiting adverse events and logistical challenges. Bispecific immune checkpoint blockers, developed to simplify the combination therapies; bispecific T-cell engagers, developed to connect the effector T-cells with tumor cells; and the next generation of CAR T-cells and the next generation of TIL therapies, developed to improve efficacy in solid tumors, are currently under clinical evaluation. This narrative review aims to summarize the current status of T-cell-directed immunotherapy, describing the brief history of development, clinical success, challenges and latest advancements that are under clinical evaluation. Evolution of monoclonal antibodies to bispecific antibodies and bispecific T-cell engagers, the latest advances in adoptive cell therapies, including the optimization of CAR T-cells for solid tumors, allogenic, universal CAR T-cells and in vivo CAR T-cell therapies are discussed in the review along with the key challenges of the therapies, such as primary and acquired resistance, limited efficacy in solid tumors, manufacturing and logistical challenges, and treatment-related toxicities.

## 1. Introduction

The therapeutic modulation of the immune system has emerged as one of the most significant advances in cancer treatment over the past two decades. Unlike conventional cytotoxic therapies, immunotherapies aim to harness and amplify endogenous immune mechanisms to recognize and eliminate malignant cells, leading in some cases to durable clinical responses and long-term survival benefits [1]. Several classes of immunotherapies, including cytokines, cancer vaccines, immune checkpoint blockers (ICBs), bispecific antibodies, chimeric antigen receptor (CAR) T-cells, and tumor-infiltrating lymphocytes (TILs), have been approved by the US FDA for the treatment of cancer, and a majority of the approvals have happened in the past 10 years. Table 1 includes the list of immunotherapy sub-classes that were introduced in the clinic along with the earliest drug approvals. Though theoretically most of the immunomodulatory drugs can target multiple immune cells, including sub-types of T-cells, natural killer (NK) cells, macrophages and dendritic cells, the effector T-cells are generally considered to be the focus for modulation. T-cell-based immunotherapies can be broadly classified into five major categories: (i) cytokine therapies that promote T-cell proliferation (e.g., interleukin-2 [IL-2]); (ii) cancer vaccines that modulate T-cell priming, activation and differentiation; (iii) immune modulators that enhance T-cell activation or reverse T-cell exhaustion, such as immune checkpoint inhibitors (ICIs); (iv) T-cell-redirecting agents, including bispecific T-cell engagers (BiTEs); and (v) adoptive cellular therapies, such as chimeric antigen receptor (CAR) T-cell therapy and tumor-infiltrating lymphocyte (TIL) therapy.

Immunostimulatory cytokines such as interleukin-2 showed the earliest signs of clinical potential for the treatment of cancer, as seen by the approval of IL-2 for the treatment of unresectable metastatic melanoma (Table 1) [2]. Though the overall response rate for IL-2 treatment was relatively low, the responses were durable in the responding patients, and the adverse events were manageable compared to chemotherapy [3,4]. However, the widespread application of cancer immunotherapy was limited until the development of immune checkpoint blocker, until ipilimumab, the anti-Cytotoxic T-Lymphocyte-Associated protein 4 (CTLA-4) monoclonal antibody belonging to the class of ICBs got approved for the treatment of unresectable metastatic melanoma [5]. The approval of ipilimumab was followed by the approval of the next sub-class of ICBs, the anti-Programmed Cell Death Protein 1 (PD-1) monoclonal antibodies nivolumab and pembrolizumab, in 2014 [6]. Anti-PD-1 monoclonal antibodies significantly improved the clinical outcomes in cancer patients and dramatically changed the treatment landscape for cancer, resulting in an explosion of research in immunotherapy. The transformative impact of immune checkpoint blockades on cancer treatment was recognized by the Nobel committee, and Dr. James Allison and Dr. Tasuku Honjo were awarded the Nobel Prize in medicine for their research on immune checkpoints in 2018 [7].

To further improve the responses and survival in patients treated with immunotherapy, combination therapy, including the combination of PD-1/PD-L1 blockers with CTLA-4 blockers, with chemotherapy and with vascular endothelial growth factor (VEGF) receptor blockers were developed. Research in the structure and functions of monoclonal antibodies resulted in the development of bispecific antibodies that were designed to target two different antigens instead of single antigen. The ability to target two different antigens by bispecific antibodies resulted in the development of bispecific T-cell engagers (BiTEs) that mainly worked by binding to both T-cells, through CD3 binding and the tumor cells, through respective target antigen binding, and facilitating the elimination of tumor cells by T-cells [8]. Blinatumomab was the first bispecific antibody targeting CD3 on T-cells and CD19 on malignant B-cells, which was approved for certain hematological cancers [9]. In addition to BiTEs, bispecific antibodies are also aimed at simplifying combination immunotherapy by targeting two different pathways, such as PD-1 and VEGF or PD-1 and CTLA-4.

The early concepts of adoptive cell therapy (ACT), involving the transfer of ex vivo cultured and expanded T-cells, was proposed in the late 1980s [10,11,12,13,14] and the research on the clinical application of ACT continued along with research on immune checkpoints [15]. However, the initial results from clinical studies were not satisfactory and the evidence for clinical efficacy was seen only after the development of second-generation CAR T-cells. CAR T-cells were first approved in 2017 for pediatric and young adult patients with acute lymphoblastic leukemia [15] and TILs were approved for metastatic melanoma in 2024 [16].

Despite these advancements, there are still significant challenges to solve. CAR T-cell and BiTE therapies are often linked to treatment-related toxicities, such as cytokine release syndrome (CRS) and immune effector cell-associated neurotoxicity syndrome (ICANS), necessitating specialized monitoring and management strategies. Additionally, the use of T-cell-based therapies in solid tumors has been constrained by tumor antigen heterogeneity, limited T-cell trafficking, and the immunosuppressive tumor microenvironment [17,18,19,20,21,22]. Manufacturing complexity, supply chain constraints, and high treatment costs further restrict broad clinical implementation of adoptive cellular therapies [23,24,25,26].

Many reviews have addressed different types of T-cell-based immunotherapies but have often focused exclusively on either ICBs or CAR T-cell therapy [23,27,28]. There are limited to no reviews offering a comprehensive synthesis encompassing monoclonal antibody-based T-cell modulation, bispecific and multispecific antibody platforms, and adoptive cellular therapies while rigorously analyzing their common mechanisms, constraints, and translational obstacles. This narrative review aims to describe the different types of T-cell-targeting cancer immunotherapies that were successfully introduced into the treatment paradigm, including ICBs, BiTEs, CAR T-cells and TILs, and briefly summarize the development history, clinical success of the class and latest ongoing clinical developments. This review intends to offer a thorough and current framework for comprehending the current status and future trajectories of T-cell-based cancer immunotherapy by integrating clinical and translational viewpoints across various therapeutic platforms. As the review did not aim to address any specific clinical- or nonclinical-related questions due to its narrative nature, literature was not collected based on pre-defined search criteria, including search terms and time period, which is required for a systematic literature review. Studies that are well-known for their impact on the research area were selected for discussion and data from publicly available sites such as FDA and clinicaltrials.gov were collected to summarize in the tables. The most recent package inserts approved by the US FDA for the respective approved drugs were used to review the current status, approved indications, dosage and regimen, safety and efficacy. Where needed simple keywords, such as bispecifics, CAR T-cells, TILs or the generic name of the drug, were used to search and obtain information.

Drugs that act by stimulating T-cell activity and proliferation and by inhibiting T-cell exhaustion, such as ICBs and bispecifics, are discussed first in the review, followed by T-cell-redirecting therapies, such as bispecific T-cell engagers, and then by adoptive cell therapies, such as CAR T-cells and TILs.

## 2. Modulators of T-Cell Activation and Exhaustion

### 2.1. ICBs

**Figure 1:**
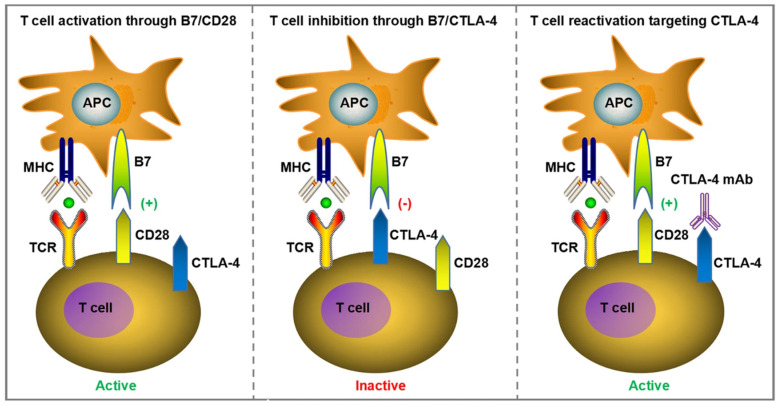
T-cell activation and CTLA-4 pathway (simplified). Reproduced from an article by Meng et al. [37]. Copyright © Meng et al. Published by *Cell Death and Disease* [37] under Creative Commons License CC.BY NC 4.0.

T-cells are thought to play a central role in the anti-tumor immune response, and their activity is regulated at multiple levels to prevent harming healthy internal organs [29]. Naïve T-cells are primed and activated in the lymph nodes and the activated T-cells are trafficked to the tumor microenvironment, where they initiate anti-tumor immune response upon interaction with tumor antigens. Activation of T-cells requires two signals, one from the presentation of an antigen peptide on the major histocompatibility complex (MHC) on the antigen-presenting cell (APC) to the T-cell receptor, and the second from the binding of a costimulatory receptor such as CD28 with B7 ligands present on APCs (Figure 1). The requirement of a second activation signal in addition to antigen presentation is an important regulatory feature of the immune response. In the absence of a second stimulatory signal, T-cells turn anergic and apoptotic, preventing the activation of T-cells in response to autoantigens [29].

Uncontrolled activation of T-cells is further regulated by the expression of inhibitory molecules on the surface of activated T-cells. The receptors and their respective ligands that are shown to regulate the immune response are commonly referred to as ‘immune checkpoints’ and the antibodies that are developed to block the immune checkpoints are referred to as ‘immune checkpoint blockers’. The discovery in the mid-1980s of pathways regulating immune response and the molecules, including receptors and their ligands, involved in fine-tuning immune cell activity dramatically changed the treatment landscape of cancer [7].

Especially, the characterization of the first immune checkpoint receptor, CTLA-4, expressed on activated T-cells, the identification of its ligands B7-H1 (CD80) and B7-H2 (CD86), expressed on APCs, and the elucidation of its role in inhibiting the costimulatory signals of T-cell activation are key milestones in tumor immunotherapy (Figure 1). The interaction between CTLA-4 and its ligands is understood to inhibit T-cell proliferation and induce anergy in the T-cells [27,28,30,31,32,33]. The discovery of CTLA-4 was closely followed by the discovery of another immune checkpoint, PD-1, and the elucidation of its role in the dampening of immune response [34,35,36]. Research on CTLA-4 and PD-1 resulted in the development of respective monoclonal antibodies blocking the inhibitory pathways to unlock anti-tumor immune responses, and the researchers Dr. James P Allison and Dr. Tasuku Honjo were awarded the Nobel Prize in medicine in 2018 for their pioneering research on CTLA-4 and PD-1 respectively [7].

**Figure 2:**
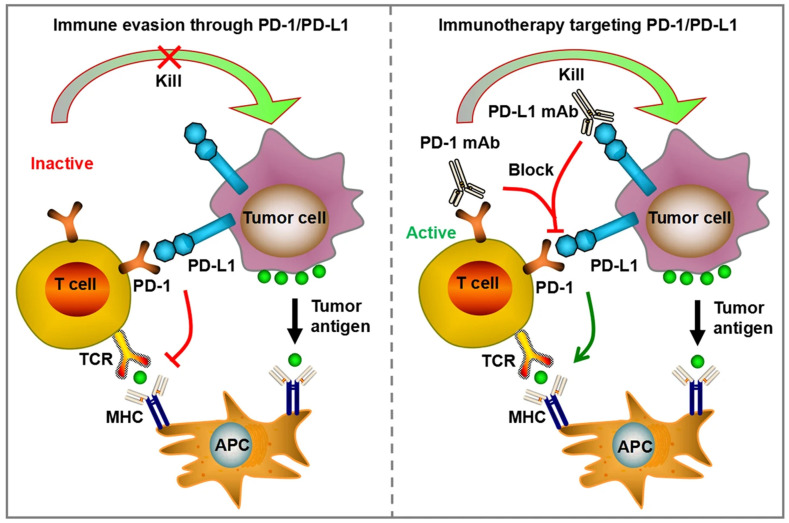
Simplified PD-1/PD-L1 pathway. Reproduced from an article by Meng et al. [37]. Copyright © Meng et al. Published by *Cell Death and Disease* [37] under Creative Commons License CC.BY NC 4.0.

The PD-1 pathway has emerged as a key pathway in the regulation of peripheral immune response. PD-1 receptors are expressed on activated T-cells and its ligand PD-L1 is broadly expressed on various cell types (Figure 2). The PD-1 pathway mainly limits uncontrolled activation of immune response and aims to protect healthy tissue from the harmful effects of immune cell activity [27,28,33]. Anti-PD-1 antibodies significantly improved the survival outcomes in cancer patients and became the backbone of treatment algorithms for a majority of cancers [37,38,39,40]. The clinical success of anti-PD-1/PD-L1 antibodies resulted in an explosion of research in immunotherapy and the clinical evaluation of multiple molecular targets for the treatment of cancer [41]. Multiple companies initiated the development of anti-PD-1 and PD-L1 antibodies as a monotherapy or as a combination therapy together with chemotherapy, other checkpoint inhibitors or targeted therapies. The number of registered clinical trials studying the efficacy and safety of anti-PD-1/PD-L1 alone or in combination therapy was estimated to be over 5000 in 2021, indicating the interest in the anti-PD-1/PD-L1 clinical development programs [42]. To date, seven anti-PD-1 and three anti-PD-L1 monoclonal antibodies have been approved by the US FDA as monotherapy or combination therapy for the treatment of various cancer sub-types (Table 2). Anti-PD-1 monoclonal antibodies have received approvals for a broad range of indications, including both solid tumors as well as hematological cancers. Key approved indications for pembrolizumab, an anti-PD-1 monoclonal antibody and its clinical outcomes are summarized in Supplementary Information Table S1. As research has progressed more cancer types have been added to the approved list of indications, and newer molecules targeting PD-1 and PD-L1 with comparatively higher response rates have been developed. A recent study in rectal carcinoma patients reported 100% complete clinical response following the treatment with the anti-PD-1 antibody dostarlimab, and the molecule was granted ‘breakthrough designation’ by the US FDA for the treatment of patients with locally advanced mismatch repair deficient (dMMR)/microsatellite instability-high (MSI-H) rectal cancer [43,44].

Biomarkers have played a significant role in the success of anti-PD-1/PD-L1 monoclonal antibodies and helped in identifying the patients with a high likelihood of response [45,46,47]. The biomarkers that were approved as companion diagnostics for use with anti-PD-1/PD-L1 monoclonal antibodies by the US FDA include PD-L1, dMMR, MSI-H, TMB, microsatellite stable (MMS or MSI-not high), EGFR and BRAF [48]. However, the impact of the biomarkers in explicitly identifying responders for therapy has been limited by factors such as tumor heterogeneity, lack of standard cut-off values, the labor-intensive nature of the techniques, extrapolation of results based on a small sample or small genomic space and the dynamic nature of the tumor [46,47]. Research is ongoing to improve the accuracy of the biomarkers and to simplify the use of diagnostic tools.

While ICBs, particularly anti-PD-1 monoclonal antibodies, have been extremely successful in the treatment of cancer, limitations to the therapy have also been reported. Resistance to ICB treatment due to the loss of tumor antigens, defects in antigen presentation, T-cell exhaustion and mutations in interferon signaling pathways resulting in the recurrence of cancer were commonly reported, along with the occurrence of autoimmunity-related side effects [49,50,51,52,53]. Lack of reliable biomarkers to predict the treatment response and challenges in the optimization of anti-PD-1 combinations with other ICBs or other molecules such as anti-vascular endothelial growth factor (VEGF) monoclonal antibodies have limited the utilization of ICBs and prompted further research.

In patients with disease progression after initial treatment with ICBs, retreatment with ICBs, termed ‘immunotherapy rechallenge’, has been proposed. While clinical evidence supporting the application of immunotherapy rechallenge is limited, early results support the use of immunotherapy rechallenge in patients who tolerated the initial therapy well and showed response [54,55,56,57]. The efficacy of immunotherapy rechallenge was found to be influenced by multiple factors, including the characteristics of patients, PD-L1 expression, cancer sub-type, monotherapy versus combination therapy and the treatment interval between initial treatment and rechallenge. Patients who had a response during initial treatment were found to likely have response during rechallenge. Similarly, patients who experienced grade 3 or higher adverse events during initial treatment were likely to have severe adverse reactions during rechallenge [57,58,59]. Careful screening of patients is recommended prior to rechallenge and confirmatory studies in larger cohorts of patients may be needed to determine the benefits of immunotherapy rechallenge in cancer patients.

To address the concerns of patient compliance and treatment logistics associated with immunotherapy combinations, the use of bispecific and trispecific antibodies was proposed. Bispecific antibodies targeting PD-1 and a second immune checkpoint, such as CTLA-4, and PD-1 and cell surface molecules involved in cancer progression, such as VEGF, have shown promising efficacy in clinical trials and are discussed in the following section.

### 2.2. Bispecific Antibodies

Monoclonal antibodies were tested and successfully introduced into clinics for the treatment of cancer long before the application of ICBs [60,61]. Monoclonal antibodies against tumor-associated antigens, such as Her2 and epidermal growth factor receptor (EGFR), and cancer-promoting cytokines, such as vascular endothelial growth factor (VEGF-A), have been approved for the treatment of multiple cancer types [61]. In addition, antibody–drug conjugates combining monoclonal antibodies with cytotoxic compounds to selectively deliver the cytotoxic compound to cancer cells via monoclonal antibodies have also been approved for the treatment of multiple cancer types [61]. The overall success of monoclonal antibodies in targeting tumor cells resulted in the evaluation of bispecific antibodies for the treatment of cancer [62,63]. Bispecific antibodies can target two different immune checkpoints (e.g., PD-1 and CTLA-4), target an immune checkpoint (e.g., PD-1) and a tumor-associated antigen (e.g., EGFR) or cytokine (e.g., VEGF), or target the T-cell receptor component CD3 and a tumor-associated antigen. The first two types of bispecific antibodies are discussed in this section, and the third category of bispecific antibodies, which mainly act by forming a link between effector T-cells and tumor cells, termed BiTEs, are discussed separately in the following section.

Bispecific antibodies designed to block two tumorigenic signaling pathways or two immune checkpoints, or to block an immune checkpoint and a tumorigenic signaling pathway, address the concerns of combination therapies, such as the logistics of drug administration and patient compliance. The list of bispecific antibodies approved by the US FDA for the treatment of cancer is presented in Table 3. Amivantamab-vmjw, a bispecific antibody against EGFR and mesenchymal epithelial transition (MET) receptor, has been approved for the treatment of adult patients with locally advanced or metastatic NSCLC with EGFR exon 20 insertion mutations [64]. Other combinations of tumorigenic signaling blockades under advanced stages (phase 2 or above) of evaluation include anti-EGFR and anti-Human Epidermal growth factor Receptor (HER) 3, and anti-EGFR [65] and anti-Leucine-rich repeat-containing G-protein-coupled receptor (LGR) 5 (Table 4) [66]. Anti-PD-1 and anti-CTLA-4 bispecific antibodies are among the most commonly evaluated dual immune checkpoint blockers in advanced stages of development [67,68,69,70]. Other dual immune checkpoint blockers include anti-PD-1 and anti-4-1BB blockers, anti-PD-1 and anti-TIGIT blockers, anti-PD-1 and anti-LAG-3 blockers, and anti-PD-1 and anti-Tim-3 blockers [63].

The combination of anti-PD-1 and anti-VEGF therapies has been shown to improve treatment outcomes in cancer patients and has been approved by US FDA for the treatment of renal cell carcinoma, NSCLC, hepatocellular carcinoma and endometrial carcinoma [71,72,73,74]. The success of the anti-PD-1/PD-L1 and anti-VEGF therapy combination encouraged the development of bispecific antibodies targeting PD-1/PD-L1 and VEGF [75,76,77,78,79,80]. Early clinical studies promising the efficacy of anti-PD-1/PD-L1 and anti-VEGF bispecific antibodies and multiple molecules are in advanced stages (phase 2 or above) of clinical development (Table 4). Anti-PD-1 and anti-TGF-β bispecific antibody is another combination of targets that has shown promising activity in preclinical studies, and multiple bispecific molecules targeting PD-1 along with TGF-β are in early clinical studies [63].

Though bispecific antibodies as a class demonstrated clinical success with multiple regulatory approvals, they are limited by emergence of on-target and off-target toxicities, development of anti-drug antibodies that can increase the elimination or inhibit the activity of the drugs and by the complexities associated with the tumor microenvironment that can impact the bioavailability of the antibodies at the target site and also inhibit the anti-tumor activity of the effector T-cells [63]. Further research on optimization of antibody components, binding affinity and avidity of the antibody, immunogenicity and pharmacokinetic properties may be needed to improve the application of bispecific antibodies in the clinical practice.

## 3. T-Cell-Redirecting Therapies

### BiTEs

**Figure 3:**
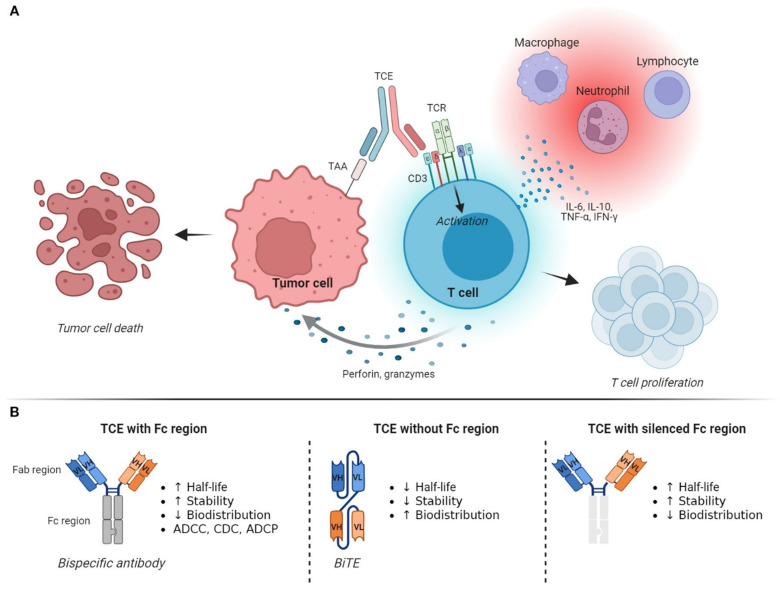
T-cell engagers: mechanism of action (**A**) and structure (**B**). Reproduced from an article by Spinazzola et al. [91]. Copyright © Spinazzola et al. Published by *Frontiers in Immunology* [91] under Creative Commons License CC.BY 4.0.

The concept of using bispecific antibodies to direct the T-cells to target tumor cells and induce tumor cell killing by forming immune synapses was introduced nearly four decades ago in the 1980s [8]. The idea of linking effector T-cells and tumor cells was optimized over time to develop the currently available bispecific antibodies. Research on monoclonal antibody structures revealed the significance of the neonatal Fc Receptor (FcRn) in the recycling of antibodies and extending the lifecycle [81], and the significance of IgG binding to Fcγ receptors on myeloid cells and natural killer (NK) cells in the activation of antibody-dependent cellular cytotoxicity (ADCC) [82,83]. As the technology of monoclonal antibodies advanced, various formats of bispecific antibodies were developed, which mainly differed based on the core structure of the molecule (IgG vs. non-IgG) and subsequent Fc-mediated functions (Figure 3) [84,85]. Bispecifics based on the IgG format retain Fc-mediated functions, have a long half-life due to FcRn-mediated recycling and are able to induce ADCC through Fcγ binding depending on the type of IgG subtype, whereas BiTEs based on the non-IgG format have higher tissue penetration but have a short half-life and lack the ability to induce antibody-induced cellular cytotoxicity [63]. Preclinical head-to-head experiments comparing the activity of IgG versus non-IgG documented the significance of the structure on the half-life and activity, and showed that the Fc portion of the molecule can determine the overall target binding property, potency and stability of the BiTE, and thereby influence the mechanism of action and cytotoxicity [86].

The approval of blinatumomab, an anti-CD19 and anti-CD3 BiTE, in 2014 for the treatment of acute lymphoblastic leukemia is considered a breakthrough in immunotherapy after ICBs, and has introduced a unique method of modulating the anti-tumor activity of effector T-cells. Blinatumomab approval triggered a wave of research on bispecific antibodies and T-cell engagers, resulting in the development and approval of several BiTE molecules for the treatment of cancer. To date, nine T-cell-redirecting bispecifics and BiTE molecules, including blinatumomab, have been approved by the US FDA for the treatment of different types of hematological cancers (Table 3). It is to be noted that almost all of the recently introduced BiTEs have been approved by the US FDA through an accelerated pathway and have received conditional approval pending confirmatory randomized controlled trials.

The characteristic feature of BiTEs is their ability to bypass TCR specificity, antigen presentation through MHC molecules and costimulatory pathways, and to induce T-cell-mediated cytotoxicity in the target cells [87]. The main advantage with the BiTE approach is that the T-cells can overcome MHC downregulation on tumor cells and effectively eliminate the target cells. Indeed, BiTEs demonstrated promising potential in improving the overall survival in certain hematological cancers [88]. A recent phase 3 study with multiple myeloma patients also demonstrated the potential of combination therapy including a BiTE in improving the survival of patients [89]. However, there has been limited success for BiTEs outside of hematological cancers and the application of BiTEs in solid tumors has been challenging due to the complex tumor microenvironment [87,90,91]. To date, two BiTEs, including tebentafusp-tebn, a bispecific fusion protein designed to connect gp100 expressing cells with CD3 of T-cells, and tarlatamab-dlle, a bispecific antibody targeting delta-like ligand (DLL) 3 on cancer cells and CD3 on T-cells, have been conditionally approved for solid tumors, including uveal melanoma and small cell lung cancer respectively (Table 3) [92,93].

The main hurdles in the clinical utilization of BiTEs include limited efficacy in solid tumors due to factors associated with the tumor microenvironment (TME) and the emergence of adverse events, such as cytokine release syndrome and infections [94]. Despite the strong rationale in preclinical studies, several BiTE molecules fail in clinical studies, and the failure is commonly attributed to pharmacokinetic properties and on-target but off-tumor toxicity [95,96]. The TME is known to be immunosuppressive and devoid of T-cells or unable to infiltrate the tumor in ‘cold’ tumors, or may consist of immunosuppressive cells, such as myeloid-derived suppressor cells (MDSCs) and regulatory T-cells (Tregs), that can inhibit the activity of effector T-cells [17,18]. Tumor stroma can also be a physical barrier for the distribution of drugs and may result in the reduced availability of BiTE molecules at the target site [19,20]. Tumor-associated antigen (TAA) heterogeneity and the presence/emergence of tumor cell clones that do not express the TAA targeted by BiTEs can also result in reduced efficacy. The use of step-up dosing strategies and increased monitoring were proposed to address the severity of CRS and infections associated with BiTEs, and has been successfully adopted into clinical practice [97]. The selection of TAAs that are abundantly expressed on tumor cells as targets for the development of BiTE molecules is considered as a promising strategy to address tumor heterogeneity. Indeed, DLL3, a transmembrane protein known to suppress the Notch signaling pathway and regulate the neural tube closure during pregnancy [98], is shown to be overexpressed in neuroendocrine and non-neuroendocrine tumors [99], and has been successfully targeted by a BiTE molecule (tarlatamab-dlle) for the treatment of small cell lung cancer (Table 3). A list of selected ongoing advanced (phase 2 or above) studies evaluating BiTE molecules is presented in Table 4. The findings from the studies can provide further guidance on strategies for the optimization of the efficacy and safety of BiTEs.

## 4. Adoptive Cell Therapies

### 4.1. CAR T-Cells

**Figure 4:**
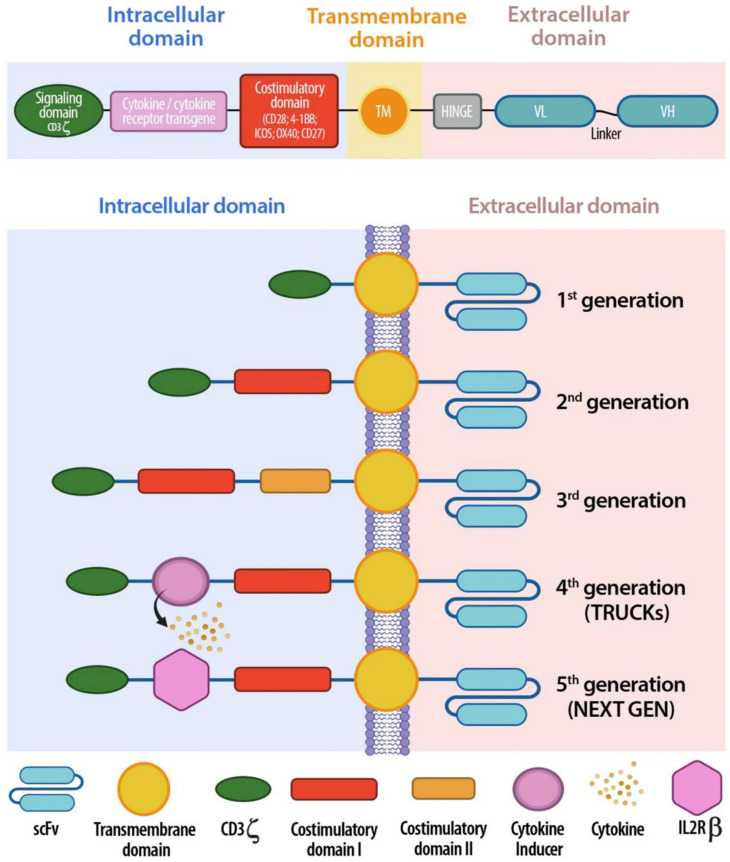
Schematic representation of CAR T-cell structure: intracellular, transmembrane, and extracellular domain (upper panel). Evolution of the five generation of CARs: from the first generation, containing only one activation domain, to the last next generation CARs, aiming to improve their safety and efficacy (lower panel). Reproduced from article by Pinto et al. [125]. Copyright © Pinto et al. Published by *Journal of Translational Medicine* [125] under Creative Commons License CC.BY 4.0.

CAR T-cells are T-cells designed to recognize specific antigens on tumor cells through their antigen-specific chimeric receptors and trigger cytotoxicity against tumor cells. The idea of chimeric T-cell receptors was first proposed in the late 1980s and involved combining variable regions (VH/VL) from the antibody with the constant regions from the T-cell receptor (TCR) [11]. The study demonstrated the feasibility of expressing chimeric receptors on T-cells and the activation of T-cells expressing chimeric receptors in response to antigens. Two years later, another team of researchers demonstrated the target antigen-dependent binding and subsequent IL-2 production and cytotoxicity of target cells using T-cells expressing chimeric receptors [13]. However, the initial design of CARs, called the ‘first generation of CAR’, which included scFv fused to the CD3ζ signaling endodomain, showed little to no anti-tumor activity in humans when evaluated in clinical studies using respective autologous CAR T-cells in patients with ovarian cancer, metastatic renal cell cancer, neuroblastoma and mantle cell lymphoma [100,101,102]. To address the concerns on clinical efficacy, the second generation of CAR T-cells designed to incorporate both stimulatory signals of T-cell activation, including TCR as well as CD28, or other costimulatory receptors, such as 4-1-BB, were evaluated [103]. The second generation of CAR thus included the CD3ζ signaling endodomain and CD28 or 4-1-BB endodomain [15]. The design of CAR further evolved to include two costimulatory endodomains, such as CD28 and 4-1-BB, along with the CD3ζ signaling endodomain, and to T-cells redirected for universal cytokine-mediated killing (TRUCKs) that secrete a cytokine, such as IL-12, upon activation. It further evolved to CARs that contain three stimulatory signals, including the CD3ζ signaling endodomain, CD28 or 4-1-BB endodomain, and a cytokine signaling endodomain (Figure 4) [23,104,105,106]. Research on the CAR structure and the resultant improvements aimed at enhancing the cytotoxic activity of the CAR T-cells and to reduce T-cell exhaustion [107,108]. Preclinical studies showed that the latest generation of CAR T-cells, including the TCR signaling domain, costimulatory domain or domains and a cytokine signaling domain or expressed proteins that blocked T-cell exhaustion ligands, improved the target specificity, increased the proliferative capacity and enhanced the cytotoxicity [109,110]. The second generation of CAR T-cells have seen clinical success, especially in patients with hematological cancers, whereas the recent iterations are still in clinical trials.

The T-cells used for the generation of CAR T-cells are usually collected from the patient (autologous) through apheresis, and genetically modified using various gene modification techniques and expanded further ex vivo before administration. Retroviral transduction is the commonly used technique for the transfer of the CAR gene into T-cells in the CAR T-cell therapies that are approved by the US FDA, whereas CAR T-cells generated using other gene modification techniques, such as electroporation, CRISPR-CAS and mRNA, are currently under evaluation [111].

The potential of CAR T-cells in providing durable responses has been documented mainly in hematological cancers, such as mantle cell lymphoma, multiple myeloma, B-cell lymphoma, including diffuse large B-cell lymphoma and follicular lymphoma, and acute lymphoblastic leukemia [112,113,114,115]. To date seven CAR T-cell therapies (all autologous T-cells) have been approved by the US FDA for hematological malignancies (Table 5). Tisagenlecleucel (Tisa-cel) was the first CAR T-cell therapy to be approved by the US FDA, and obecabtagene autoleucel (obe-cel) was the most recently approved CAR T-cell therapy (Table 5). Autologous CAR T-cell therapies have shown encouraging potential in clinical trials as well as real-world studies, with documented responses in the majority of patients and improvements in progression-free and overall survival in responding patients after a single dose of CAR T-cell therapy [116,117,118,119,120,121]. CAR T-cell therapies are also associated with serious adverse effects, such as cytokine release syndrome (CRS), neurotoxicity, prolonged cytopenia and recurrent infections [115]. Recently, rare cases of second primary malignancies of T-cell origin were reported in patients treated with CAR T-cell therapies [122,123]. However, the benefits of CAR T-cell therapy outweigh the risks associated with the treatment and better algorithms are now available for the management of serious adverse events in patients treated with CAR T-cell therapy.

While autologous CAR T-cell therapies have shown promising clinical efficacy, their utilization has been limited by manufacturing and supply chain challenges [23]. To address the expected time delays associated with the manufacturing and shipping of autologous CAR T-cells, allogeneic/donor-T-cell-derived CAR T-cell therapies are proposed. Though the concept of allogeneic CAR T-cell therapy is interesting, the approach has the risks of ‘graft versus host disease’, which occurs when the T-cell receptors present on the allogeneic CAR T-cell surface recognize the antigens on the surface of patient’s healthy tissues and trigger immune response, and ‘host versus graft disease’, which occurs when the patient’s immune system rejects the allogeneic CAR T-cells. The former reaction can be serious and life-threatening, whereas the latter reaction can lead to treatment failure [24,25,26]. To address the concerns of GvHD and HvG, multiple approaches such as selecting T-cells with low TCR signaling during manufacture, knocking out of TCR and CD52 from T-cells, and expressing CD47 on T-cells have been proposed [24]. The optimized allogeneic CAR T-cells with reduced GvHD and HvG potential are currently under advanced stages of clinical evaluation. The list of key allogeneic CAR T-cell therapies under clinical evaluation is presented in Table 6. Interestingly, despite the promising potential to address manufacturing delays associated with autologous CAR T-cells, progress in allogeneic CAR T-cells is relatively slow and most studies are in early-phase trials. Results from clinical studies are needed to support the continued interest in allogeneic cell therapies [124].

Ongoing research is also focused on developing CAR T-cell therapies for solid tumors, which has been elusive despite the success in hematological cancers. Utilization of CAR T-cell therapies for solid tumors has been challenging mainly due to difficulties in identifying the tumor-specific target antigen, immunosuppressive tumor microenvironment, restricted T-cell chemotaxis and also due to the occurrence of severe toxicity associated with CAR T-cells in solid tumors [21]. Recently multiple antigens, such as claudin (CLDN) 18.2, EGFR, CD70, and mesothelin, have been shown to be viable targets for the utilization of CAR T-cell therapies, and multiple approaches to overcome the extreme conditions in tumor microenvironment, such as armoring CAR T-cells to express stimulatory cytokines including IL-2, IL-12 and IL-15, and expressing proteins to block T-cell exhaustion pathways have been proposed [126,127]. The list of key phase 1/phase 2 studies evaluating CAR T-cells in solid tumors is summarized in Table 7. Among the studies that reported clinical results, satricabtagene autoleucel (satri-cel)/CT041, an autologous CAR T-cell therapy targeting CLDN18.2, has shown manageable safety and promising efficacy in early clinical trials in patients with gastrointestinal cancers and has received Regenerative Medicine and Advanced Therapeutics (RMAT) designation from the US FDA [128,129,130]. However, it is to be noted that the clinical evidence generated to date is from early-phase trials and randomized phase 3 studies are needed to validate the findings from the studies. Secondly, while the outcomes are promising, the efficacy seen with solid tumor indications is still not comparable to the efficacy in hematological malignancies. Further research is needed to improve the efficacy of CAR T-cells in solid tumor indications.

Currently approved ex vivo CAR T-cell therapies are limited by manufacturing process and supply chain complexities and high costs. To address the concerns associated with manufacturing process, an in vivo CAR T-cell approach was proposed [131]. The in vivo CAR T-cell strategy involves the generation of CAR T-cells from T-cells within the patient by delivering the CAR gene to the patient through gene editing tools, such as viral vectors, mRNA and CRISPR technologies. In vivo CAR T-cell therapies are in the early stages of development compared to allogeneic and next-gen CAR T-cell therapies, and the interest in the clinical development of CAR T-cell therapies has increased recently [125,131]. Major challenges that need to be addressed in the development of in vivo CAR T-cell therapies include the development of delivery methods (e.g., viral vectors and nanocarriers) for CAR transgenes that are stable and have adequate bioavailability at the target site, and which also selectively and effectively transfect T-cells in the body [131]. Insertional mutagenesis risk is another concern with viral vectors that would need long-term monitoring of the patients for the development of secondary malignancies [132]. If successful in addressing the challenges, in vivo CAR T-cell therapies have the potential to have a paradigm-shifting impact on the treatment of cancer. The list of selected biotech companies developing in vivo CAR T-cell therapies is summarized in Table 8.

### 4.2. TILs

**Figure 5:**
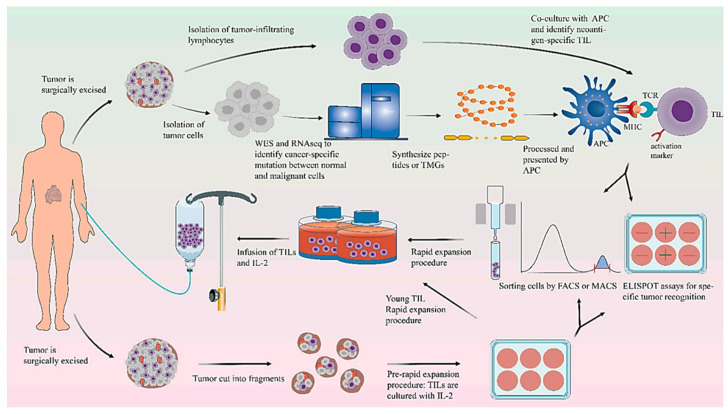
General scheme of preparation for TILs. Reproduced from an article by Zhao et al. [133]. Copyright © Zhao et al. Published by *Cancers* [133] under Creative Commons License CC.BY 4.0.

TIL therapy involves the isolation of physiologically infiltrating lymphocytes from the tumor tissues, expanding the isolated lymphocytes in vitro and infusing them back into the patients along with cytokines, such as IL-2, typically following lymphodepletion therapy similar to CAR T-cell therapy (Figure 5) [133,134]. The concept of using lymphocytes infiltrating the tumors to treat cancer was first proposed in 1986 [10]. Using a murine MC-38 colon adenocarcinoma model, researchers showed that the treatment of tumor-bearing mice with TILs and cyclophosphamide combination resulted in the elimination of liver and lung metastatic deposits, and that the addition of IL-2 to the combination of TILs and cyclophosphamide resulted in the complete elimination of liver metastatic deposits in 100% of mice and lung metastatic deposits in 50% of mice [10]. The team continued the research and extended their experience from murine models to clinical trials in melanoma patients, showing the feasibility of extracting lymphocytes from freshly resected tumors, expanding them in vitro and reinfusing them into patients [135]. The study reported objective responses in 9 out of 15 IL-2 treatment-naïve patients, and in 2 out 5 patients who previously failed IL-2 therapy and regression of tumors lungs, liver, bone, skin and subcutaneous sites. The response reportedly lasted for 2–13 months in responding patients [135].

TIL therapy is thought to be advantageous over CAR T-cell therapy, especially in solid tumors where tumor heterogeneity is a challenge. TILs have the ability to target multiple antigens, increasing the likelihood of tumor killing in solid tumors. TILs are also primed specifically for the neoantigens of the tumors, reducing the possibility of ‘off-tumor’ cytotoxicity, and are superior to CAR T-cells in their ability to infiltrate the tumor microenvironment due to the expression of chemokine receptors on their surface. The risk of severe and life-threatening cytokine release syndrome seen with CAR T-cell therapy is also low with TIL therapy [136,137,138]. Clinical research on the utilization of TILs for the treatment of cancers continued for nearly two decades and led to the approval of lifileucel in 2024 for the treatment of metastatic melanoma, which was previously treated with a PD-1 blocking antibody and, if BRAF V600-positive, a BRAF inhibitor with or without a MEK inhibitor [139].

While TIL therapy has advantages over CAR T-cell therapy, it is limited by similar manufacturing and supply chain-related challenges as seen with CAR T-cell therapy. In addition, the quality of TILs extracted is dependent on the tumor tissue used for extraction and can result in an inconsistent quality of the product. Secondly the TIL product is heterogenous and may include lymphocytes that are not primed for tumor tissues and include exhausted T-cells [139]. Furthermore, TIL therapy is not recommended for all patients, and the patients are carefully selected based on the expected quality of TILs in the tumors, the overall condition and immune status of the patient and the heterogeneity of the tumors [139]. The latest research is aimed at addressing the concerns associated with TIL therapy, and novel approaches, such as using genetically modified TILs that can secrete IL-15 to stimulate T-cell proliferation and sorting TILs based on specific T-cell markers to select the most effective T-cells, have been proposed [139]. Multiple clinical studies have been initiated to evaluate the next generation of TIL therapies and are in the early stages of clinical research (Table 9). Clinical evidence from randomized controlled trials is needed to conclude the benefits of TIL therapies in other cancer subtypes. Findings from ongoing early-stage studies could guide the design of advanced phase 3 clinical studies and eventually the approval of additional TIL therapies.

## 5. Conclusions

Targeting T-cell-mediated immunity has fundamentally transformed cancer therapy and represents one of the most significant advancements in contemporary oncology. Among T-cell-based strategies, the approach of targeting the T-cell activation and T-cell exhaustion, especially through the inhibition of the PD-1/PD-L1 axis, has attained the most extensive clinical success and is now firmly recognized as a standard element of treatment for various malignancies (Table 10). Anti-PD-1/PD-L1 blockers as monotherapy or as combination therapy demonstrated efficacy in several solid tumors and heme malignancies and improved patient survival (Table 10). However, the approach of targeting T-cell activation and exhaustion has been limited by the incidence of treatment-limiting adverse events and lack of response in a significant proportion of patients. The latest iterations of bispecific antibodies aiming to improve response rates demonstrated success in NSCLC and need to be evaluated in other cancer types (Table 10). T-cell-redirecting therapies such as BiTEs have the benefits of showing cytotoxic activity independent of MHC restriction and avoiding concerns of manufacturing and supply chain issues (Table 10). While most of the BiTEs have been successful in hematological malignancies, two BiTEs have shown activity in solid tumor indications, such as metastatic melanoma and SCLC. However, it is to be noted that the majority of BiTE molecules have only received conditional approval and randomized clinical trials are needed to confirm their efficacy and safety. Also, while the incidence of CRS and ICANS is comparatively low, patients need to be carefully monitored for adverse events such as infections, and labels for all BiTE molecules carry a ‘black box’ warning for severe and life-threatening CRS and ICANS (Table 11). Cell therapies, including CAR T-cell therapy and TIL therapy, aim to personalize the treatment and improve the response rates in patients. CAR T-cell therapy was successful in improving response rates, and a majority (and in some cases almost all) of the treated patients showed objective responses, which deepened over time and were durable. However, the efficacy of CAR T-cells has been limited to hematological malignancies, and clinical success in solid tumors is still awaited. Secondly, CAR T-cell therapy is associated with incidences of CRS and ICANS, which can be severe and life-threatening, prolonged cytopenias and secondary T-cell malignancies, which are highlighted through a ‘black box’ warning on the labels (Table 11). TIL therapy, on the other hand, demonstrated efficacy in metastatic melanoma patients refractory to ICB treatment and improved patient survival, but carries risk profile and ‘black box’ warning similar to CAR T-cell therapy (Table 11).

It is to be noted that all therapeutic classes of immunotherapies discussed in this review have been studied in carefully designed clinical trials. The inclusion and exclusion criteria for the clinical trials vary widely across the trials and the patients are screened based on specific biomarker criteria. For that reason, it is not possible to make cross-trial comparisons or draw conclusions on the comparative efficacy of the therapeutic classes. Though propensity-matched scores are introduced to account for the differences in selection criteria, accurate comparative claims can only be made when appropriately powered head-to-head RCTs are conducted. This review does not intend to compare the efficacy and safety across therapeutic classes, but to describe the current clinical status of the T-cell-targeting classes and discuss the latest advances within.

Despite the achievements and improvement in anti-tumor activity, the clinical benefit of T-cell-targeting therapies remains uneven across patient populations and cancer types. Further research is needed to develop the next iterations of the therapies. Among the latest advancements within the therapeutic classes, bispecific and multispecific antibody platforms, including dual immune checkpoint and immune checkpoint–angiogenesis combinations, represent an evolving class of therapies designed to simplify combination regimens and improve anti-tumor efficacy. Bispecific antibodies and BiTEs are in phase 3 clinical trials designed to confirm the clinical efficacy and safety and provide rationale for regulatory approval. Next-gen CAR T-cell therapies developed to enhance efficacy in solid tumors are in early stages of development, where the safety of the product is evaluated along with preliminary evidence of efficacy. Additional time is needed to optimize the next-gen CAR T-cell therapy and move to the confirmatory phase 3 trials. Similarly, allogeneic CAR T-cell products are still in phase 1 and early phase 2 trials, and evidence is awaited to demonstrate that the depth and durability of responses from allogeneic CAR T-cell therapies are on par with autologous CAR T-cell therapies. In vivo CAR T-cell therapies are promising and generating excitement across the research community for their potential to address several manufacturing and supply chain concerns seen with CAR T-cell therapy. However almost all of the in vivo CAR T-cell products are either in the preclinical/investigative new drug (IND) application enabling stage or early phase 1 dose finding/first-in-human (FIH) stage, and the results from the clinical trials may provide evidence for the feasibility of in vivo therapy. In conclusion, new T-cell-targeting therapies may be introduced into the clinic in the coming years and further improve the prognosis of cancer patients.

## Figures and Tables

**Table 1:** Classes of immunotherapies and their earliest FDA approval.

Class	Sub-Class	Drugs	Earliest Approval (Year)
Cytokines	IL-2	Aldesleukin	May 1992
Cancer vaccines	DC vaccine	Sipuleucel-T	April 2010
ICBs	CTLA-4 blockers	Ipilimumab	March 2011
	PD-1 blockers	Pembrolizumab	September 2014
	LAG-3 blockers	Relatlimab *	March 2022
Bispecifics	BiTEs (CD3 and CD19)	Blinatumomab	December 2014
CAR T-cells	CD19	Tisagenlecleucel	August 2017
TILs	TILs	Lifileucel	February 2024

* Relatlimab was approved as combination therapy with nivolumab.

**Table 2:** List of anti-PD-1 and anti-PD-L1 antibodies approved by the US FDA for the treatment of cancer.

Trade Name	Drug	Company	Target	First Approval
Keytruda	Pembrolizumab	Merck, Rahway, NJ, USA	PD1	September 2014
Opdivo	Nivolumab	Bristol-Myers Squibb, Princeton, NJ, USA	PD1	December 2014
Tecentriq	Atezolizumab	Genentech/Roche, Basel, Switzerland	PD-L1	May 2016
Bavencio	Avelumab	Pfizer, New York, NY, USA	PD-L1	March 2017
Impfinzi	Durvalumab	AstraZeneca, Cambridge, UK	PD-L1	May 2017
Libtayo	Cemiplimab	Regeneron, Tarrytown, NY, USA	PD1	September 2018
Jemperli	Dostarlimab	GlaxoSmithKline, London, UK	PD1	April 2021
Zynz	Retifanlimab	Incyte, Wilmington, DE, USA	PD1	March 2023
Loqtorzi	Toripalimab	Coherus Biosciences, Redwood City, CA, USA	PD1	October 2023
Tevimbra	Tislelizumab	BeOne *, Basel, Swizerland	PD1	March 2024

* Formerly known as BeiGene.

**Table 3:** List of bispecific antibodies, including BiTEs, approved by the US FDA for the treatment of cancer.

Trade Name	Drug	Target	Company	First Approval
Blincyto	Blinatumomab	CD3 & CD19	Amgen, Thousand Oaks, CA, USA	December 2014
Rybrevant	Amivantamab-vmjw	EGFR & MET	Johnson & Johnson, New Brunswick, NJ, USA	May 2021
Kimmtrak	Tebentafusp-tebn	gp100 & CD3	Immunocore, Oxfordshire, UK	January 2022
Tecvayli	Teclistamab-cqyv	BCMA & CD3	Johnson & Johnson, New Brunswick, NJ, USA	October 2022
Lunsumio	Mosunetuzumab-axgb	BCMA & CD3	Genentech/Roche, Basel, Switzerland	December 2022
Epkinly	Epcoritamab-bysp	CD20 & CD3	Abbvie, North Chicago, IL, USA	May 2023
Columvi	Glofitamab-gxbm	CD20 & CD3	Genentech/Roche, Basel, Switzerland	June 2023
Talvey	Talquetamab-tgvs	GPRC5D & CD3	Johnson & Johnson, New Brunswick, NJ, USA	August 2023
Elrexfio	Elranatamab-bcmm	BCMA & CD3	Pfizer, New York, NY, USA	August 2023
Imdelltra	Tarlatamab-dlle	DLL3 & CD3	Amgen, Thousand Oaks, CA, USA	May 2024

**Table 4:** List of select clinical studies evaluating bispecific antibodies (including BiTEs).

Lead Molecule	Target	Target Population	Sponsor	Clinical Trial Stage
SI-B001	EGFR & HER3	Multiple solid tumors	Sichuan Baili Pharmaceutical Co., Ltd., Chengdu, China	Phase 2
Petosemtamab (MCLA-158)	EGFR & LGR5	Multiple solid tumors	Merus N.V., Utrecht, The Netherlands	Phase 3
HS-20117	EGFR & MET	Non-squamous NSCLC	Hansoh BioMedical R&D Company, Jiangsu, China	Phase 2/3
AK104	PD-1 & CTLA-4	Pancreatic cancer	Akeso, Guangdong, China	Phase 2
Volrustomig	PD-1 & CTLA-4	Multiple solid tumors	AstraZeneca, Cambridge, UK	Phase 3
Vudalimab	PD-1 & CTLA-4	Colorectal cancer	Xencor, Inc., Pasadena, CA, USA	Phase 2
KN046	PD-L1 & CTLA-4	NSCLC	Jiangsu Alphamab Biopharmaceuticals Co., Ltd., Jiangsu, China	Phase 2
Acasunlimab	PD-L1 & 4-1-BB	NSCLC	Genmab, Copenhagen, Denmark	Phase 2
Rilvegostomig	PD-1 & TIGIT	Multiple solid tumors	AstraZeneca, Cambridge, MA, USA	Phase 3
Ivonescimab	PD-1 & VEGF	NSCLC	Summit Biotherapeutics, Miami, FL, USA	Phase 3
SSGJ-707	PD-1 & VEGF	Multiple solid tumors	Sunshine Guojian Pharmaceutical (Shanghai) Co., Ltd., Shanghai, China/Pfizer, New York, NY, USA	Phase 2
B1962	PD-L1 & VEGF	Colorectal cancer	Tasly Biopharmaceuticals Co., Ltd., Tianjin, China	Phase 2
BNT327/Pumitamig	PD-L1 & VEGF-A	SCLC	BioNTech SE Mainz, Germany & BMS, Princeton, NJ, USA	phase 2/3
Ubamatamab	MUC-16 & CD3	Ovarian cancer	Regeneron Pharmaceuticals, Tarrytown, NY, USA	Phase 2
Linvoseltamab	BCMA & CD3	Multiple Myeloma	Regeneron Pharmaceuticals, Tarrytown, NY, USA	Phase 2

**Table 5:** List of CAR T-cell therapies approved by the US FDA.

Trade Name	Drug	Target	Company	First Approval
Kymriah	Tisagenlecleucel	CD19	Novartis, Basel, Switzerland	August 2017
Yescarta	Axicabtagene ciloleucel	CD19	Gilead/Kite, Foster City, CA, USA	October 2017
Tecartus	Brexucabtagene autoleucel	CD19	Gilead/Kite, Foster City, CA, USA	July 2020
Breyanzi	Lisocabtagene maraleucel	CD19	Bristol-Myers Squibb, Princeton, NJ, USA	February 2021
Abecma	Idecabtagene vicleucel	BCMA	Bristol-Myers Squibb, Princeton, NJ, USA	March 2021
Carvykti	Ciltacabtagene autoleucel	BCMA	Johnson & Johnson, New Brunswick, NJ, USA Legend Bio, Somerset, NJ, USA	February 2022
Aucatzyl	Obecabtagene autoleucel	CD19	Autolus Therapeutics, London, UK	November 2024

**Table 6:** List of select allogeneic CAR T-cell therapies in clinical development.

Lead Molecule	Antigen Target	Company	Stage	Target Population
Cemacabtagene ansegedleucel (cema-cel)	CD19	Allogene Therapeutics, South San Francisco, CA, USA	Phase 2	First line LBCL
Lasmecabtagene timgedleucel (lasme-cel)	CD22	Cellectis, Paris, France	Phase 1	B-cell ALL
Etivelcabtagene erigedleucel (eti-cel)	CD20 and CD22 dual	Cellectis, Paris, France	Phase 1	NHL
FT825	HER2	Fate Therapeutics, San Diego, CA, USA	Phase 1	Solid tumors
P-BCMA-ALL-01	BCMA	Poseida Therapeutics, San Diego, CA, USA	Phase 1	MM
P-CD19CD20-ALL-01	CD19 and CD20 dual	Poseida Therapeutics, San Diego, CA, USA	Phase 1	B-cell malignancies
CB-010	CD19	Caribou Biosciences, Berkeley, CA, USA	Phase 1	NHL
CB-011	BCMA	Caribou Biosciences, Berkeley, CA, USA	Phase 1	MM
CAR NK	BCMA	Legend Bio, Somerset, NJ, USA	Phase 1	MM
CAR ab T-cells	CD20	Legend Bio, Somerset, NJ, USA	Phase 1	NHL
CAR gd T-cells	CD19 and CD20 dual	Legend Bio, Somerset, NJ, USA	Phase 1	NHL

**Table 7:** List of select CAR T-cell products under clinical evaluation for solid tumors.

Company	CAR T-Cell Product	Target Patients	Phases
TCRCure Biopharma Ltd. Los Angeles, CA, USA	Anti-ALPP CAR T-cells	Solid tumor	Early phase 1
Chengdu Ucello Biotechnology Co., Ltd. Chengdu, China	Anti-claudin 18.2 CAR T-cell	Claudin18.2-positive advanced solid tumors	Early phase 1
Tcelltech Inc., Mannheim, Germany	TX103 (anti-B7-H3 CAR T-cell)	Solid tumors	Phase 1
Chongqing Precision Biotech Co., Ltd., Chongqing, China	Anti-CD70-targeted CAR T-cells	RCC; lung cancer; anaplastic thyroid carcinomas; ovarian cancer; cervical cancer; thymic carcinoma	Phase 1
Chongqing Precision Biotech Co., Ltd., Chongqing, China	Anti-CEA-targeted CAR T-cells	Gastrointestinal cancers, including gastric, colon, rectal, pancreas and esophagus cancer; cholangiocarcinoma; breast cancer; lung cancer	Phase 1/2
Poseida Therapeutics, Inc., San Diego, CA, USA	Anti-P-MUC1C-ALLO1 CAR T-cells	Breast cancer; ovarian cancer; NSCLC; CRC; pancreatic cancer; RCC; nasopharyngeal cancer; HNSCC and gastric cancer	Phase 1
A2 Biotherapeutics Inc., Agoura Hills, CA, USA	A2B694	CRC, NSCLC; pancreas cancer; ovarian cancer; mesothelioma	Phase 1/2
A2 Biotherapeutics Inc., Agoura Hills, CA, USA	A2B530	CRC, NSCLC; pancreas cancer; and metastatic cancer	Phase 1/2
A2 Biotherapeutics Inc., Agoura Hills, CA, USA	A2B395	CRC, NSCLC, HNSCC; RCC; kidney cancer; and TNBC	Phase 1/2
Suzhou Immunofoco Biotechnology Co., Ltd., Jiangsu, China	Anti-claudin 18.2 CAR T-cell	Gastric cancer; pancreatic cancer; ovarian cancer and gastroesophageal junction adenocarcinoma	Phase 1
CARsgen Therapeutics Co., Ltd., Shanghai, China	CT041	Gastric adenocarcinoma; pancreatic cancer; gastroesophageal Junction adenocarcinoma	Phase 1/2
BioNTech Cell & Gene Therapies GmbH, Mainz, Germany	Anti-CLDN6 CAR T-cell	Solid tumor	Phase 1
CytoMed Therapeutics Pte Ltd., Singapore	Allogeneic NKG2DL-targeting Chimeric Antigen Receptor-grafted CAR T-cells	Relapsed/refractory cancer	Phase 1
Fate Therapeutics, San Diego, CA, USA	FT825	Advanced solid tumor	Phase 1
Fate Therapeutics, San Diego, CA, USA	FT836	NSCLC; CRC; breast cancer; ovarian cancer; endometrial cancer; HNSCC	Phase 1

**Table 8:** List of biotech companies developing in vivo CAR T-cell therapies.

Company	Partner	Antigen Target	Delivery Platform	Development Stage	Therapeutic Area Focus
Interius Biotherapeutics, Philadelphia, PA, USA	Gilead	CD20	Lentivirus	Phase 1	Heme malignancies and autoimmune disorders
EsoBiotec, Mont-Saint-Guibert, Belgium	AstraZeneca	BCMA	Lentivirus	Phase 1	Heme malignancies and autoimmune disorders
Umoja Biopharma, Seattle, WA, USA	Abbvie	CD19; CD20 & CD22	Lentivirus	Phase 1	Heme malignancies and autoimmune disorders
Capstan, San Diego, CA, USA	Abbvie	CD19; BCMA	LNP	Phase 1	Autoimmune disorders
Vyriad, Rochester, Minnesota	Novartis	Not disclosed	Lentivirus	Not disclosed	Autoimmune disorders
Kelonia, Boston, MA, USA	Astellas	BCMA	Lentivirus	Preclinical	Heme malignancies and autoimmune disorders
Tidal Therapeutics *, Cambridge, MA, USA	Sanofi *	Not disclosed	Not disclosed	Not disclosed	Not disclosed
Orna Therapeutics, Watertown, MA, USA	Simnova	CD19	LNP	Preclinical	Autoimmune
Tessera, Somerville, MA, USA	None	Not disclosed	LNP	Preclinical	Autoimmune
Orbital Therapeutics, Cambridge, MA, USA	None	CD19	LNP	Preclinical	Autoimmune
Legend Bio, Somerset, NJ, USA	None	Not disclosed	Not disclosed	Preclinical	Undisclosed
PersonGen Biotherapeutics, Jiangsu, China	None	CD19	Not disclosed	Phase 1	Heme malignancies

LNP, lipid nanoparticles. * Tidal therapeutics got acquired by Sanofi in 2021.

**Table 9:** Early clinical trials evaluating TIL therapies for cancer.

Company	Lead Molecule	Stage	Target Population
Iovance Biotherapeutics, San Carlos, CA, USA	Lifileucel	Phases 1–2	NSCLC, HNSCC, melanoma
Obsidian Therapeutics, Cambridge, MA, USA	OBX115	Phases 1–2	Melanoma, NSCLC
AgonOx, Inc., Portland, OR, USA	AGX-148	Phase 1	Solid tumors
BioSyngen Pte Ltd., Singapore	BRG-01	Phase 2	Nasopharyngeal carcinoma
BioSyngen Pte Ltd., Singapore	BRL-03	Phase 1	Solid tumors including lung
BioSyngen Pte Ltd., Singapore	BST-02	Phase 1	Liver cancer
BioSyngen Pte Ltd., Singapore	BGT07	Phase 1	Multiple solid tumors
Grit Biotechnology, Shanghai, China	GT-201	Phase 1	Multiple solid tumors
Grit Biotechnology, Shanghai, China	GTE-001	Phase 1	Lung adenocarcinoma
Hervor Therapeutics, Zhejiang Sheng, China	undisclosed	Phase 1	Solid tumors
Instil Bio, Dallas, TX, USA	ITIL-206	Phase 1	Solid tumors
Intima Bioscience, Inc., New York, NY, USA	CISH knock out TIL	Phase 1	Gastrointestinal cancers
Shanghai Juncell Therapeutics, Shanghai, China	GC-203	Phase 1	Solid tumors
	GC-101	Phase 1	NSCLC, melanoma, solid tumors
Suzhou BlueHorse Therapeutics Co., Ltd., Jiangsu, China	LM-103	Phase 1	Solid tumors

**Table 10:** Summary of key benefits and drawbacks seen with immunotherapy sub-classes *.

Class	Sub-Class	Key Benefits	Key Drawbacks	References
ICBs	Anti-CTLA-4	- Durable long-term response and improved overall survival - Use in PD-1 blocker combination	- Low overall response rates - Severe and sometimes life-threatening toxicities	[140,141,142,143]
	Anti-PD-1	- Efficacy demonstrated in several indications, including solid tumors - Improved efficacy with combination therapy - Durable long-term response - Comparatively favorable profile	- Incidence of immune-related adverse events - Incidence of treatment-limiting adverse events leading to discontinuation of therapy	[144,145,146,147]
	Anti-LAG-3	- Improved efficacy outcomes, such as PFS when combined with nivolumab - Combination approved as a single infusion formulation - Favorable safety profile compared to ipi combination	- Approved only as combination therapy - Data on OS benefits not available to date - Unfavorable risk profile compared to nivo monotherapy with approximately 12% treatment discontinuations due to any grade AE	[148,149,150,151]
BiTEs	Anti-CD3 and CD19 Anti-CD3 and CD20 Anti-CD3 and BCMA Anti-CD3 and GPRC5D	- Compared to CAR T-cells, CD19 and BCMA BiTEs are available off-the-shelf and do not have manufacturing and supply chain issues - Comparatively lower incidence of CRS and ICANS - Ability to induce target activity independent of MHC restriction - Lymphodepletion is not required	- Response rates lower compared to CAR T-cells - BiTEs with shorter half-life (~2 h) may require continuous infusion - Incidence of infection with anti-BCMA and anti-CD20 antibodies - While the incidence and severity of CRS and ICANS is comparatively low, the risk is not negligible and is emphasized as ‘black box’ warning	[152,153,154,155,156,157,158,159,160]
	Anti-CD3 and DLL3	- Effective in solid tumor indications - Deep clinical benefit in a subset of patients	- Incidence of CRS and ICANS and included as ‘black box’ warning - Only a fraction of the patients respond to therapy	[93,161,162]
	Anti-CD3 and GP100	- Overall survival significantly improved in patients	- Indication approved only for uveal melanoma - ‘Black box’ warning for life-threatening CRS - Relatively modest response rate	[163,164,165,166]
Bispecific antibody	Anti-EGFR and MET	- Efficacy seen in solid tumors - Manageable safety - No ‘black box’ warning at the time of reporting - Approved as monotherapy and as combination therapy for first-line treatment	- Cytopenias seen in combination with chemotherapy - Venus thromboembolism seen in combination with Lazertinib - Approved only for NSCLC indication	[64,167,168,169,170]
CAR T-cell therapy	Anti-CD19 Anti-BCMA	- Responses seen in majority patients or in almost all patients in case of anti-BCMA CAR T-cells - Single one-time treatment, which may result in deep durable responses - Improvements in survival rates seen - Real-world efficacy similar to clinical trials	- Limited efficacy in solid tumors - Black box warning includes: - Risk of severe and life-threatening CRS and ICANS - Risk of cytopenia - Risk of secondary T-cell malignancies - Manufacturing and supply chain concerns - Treatment costs - Limited number of treatment centers	[157,159,171,172,173,174,175,176,177,178,179,180]
TIL therapy	Anti-MAGE	- Responses seen in patients relapsing after PD-1 therapy - Single one-time treatment, which may result in deep durable response	- Boxed warning includes treatment-related mortality, prolonged severe cytopenia, severe infection, cardiopulmonary and renal impairment - Manufacturing and supply chain concerns - Full approval is contingent on confirmatory phase 3 trial - Approved only for melanoma	[139,181,182]

* List presented here is a general summary and should not be used for patient management. Refer to respective package inserts for up-to-date information on adverse events seen in the patients and to latest guidelines from expert consensus recommendations for management of adverse events.

**Table 11:** Summary of key adverse events and management recommendations *.

Class	Sub-Class	Key Adverse Events	Management	References
ICBs	Anti-CTLA-4 Anti-PD-1 Anti-LAG-3	- Immune related AEs (irAEs) such as diarrhea and colitis - Pyrexia, rash, pneumonitis, hepatitis, endocrine dysfunction, myocarditis, skin reactions	- Routine monitoring and treatment discontinuation - Use of corticosteroids or immunosuppressants	[183,184,185]
BiTEs	Anti-CD3 and CD19 Anti-CD3 and CD20 Anti-CD3 and BCMA Anti-CD3 and GPRC5D Anti-CD3 and DLL3 Anti-CD3 and GP100	- CRS, ICANS, infections cytopenias	- Routine monitoring and in some cases, hospitalization is required for monitoring - Step-up dosing - Use of prophylactic antibiotics and antivirals	[186]
Bispecific antibody	Anti-EGFR and MET	- Infusion-related reactions - Rash, paronychia, and pruritus (possibly due to EGFR blockade) - Peripheral edema and hypoalbuminemia (possibly due to MET blockade) - Cardiovascular events including venus thromboembolism	- Regular monitoring for signs of skin and cardiovascular events	[168,169,170]
CAR T-cell therapy	Anti-CD19 Anti-BCMA	- Severe and life-threatening CRS and ICANS - Prolonged cytopenias - Infections	- Monitoring for CRS and ICANS is mandated; in some cases, hospitalization is required for monitoring - Prophylactic use of tocilizumab, corticosteroids and, in some cases, antibiotics - Long term monitoring for secondary T-cell malignancies	[187,188,189,190]
TIL therapy	Anti-MAGE	- Treatment-related mortality, prolonged severe cytopenia, severe infection, cardiopulmonary and renal impairment	- Careful selection of patient eligibility for treatment - Monitoring for immune-related, infusion-related and IL-2 related toxicity - Prophylactic use of antibiotics, antifungals and antivirals - Use of tocilizumab with or without corticosteroids as needed	[191,192]

* List presented here is a general summary and should not be used for patient management. Refer to respective package inserts for up-to-date information on adverse events seen in the patients and to latest guidelines from expert consensus recommendations for management of adverse events.

## Data Availability

No new data were created or analyzed in this study.

## Supplementary Materials

The following supporting information can be downloaded at: https://www.mdpi.com/article/10.3390/biomedicines14030654/s1, Table S1. Efficacy outcomes reported for pembrolizumab (Pembro; PD-1 blocker).

## References

[1] Yang Y. (2015). Cancer Immunotherapy: Harnessing the Immune System to Battle Cancer. J. Clin. Investig..

[2] Rosenberg S.A. (2014). IL-2: The First Effective Immunotherapy for Human Cancer. J. Immunol..

[3] Atkins M.B., Kunkel L., Sznol M., Rosenberg S.A. (2000). High-Dose Recombinant Interleukin-2 Therapy in Patients with Metastatic Melanoma: Long-Term Survival Update. Cancer J. Sci. Am..

[4] Dutcher J.P., Schwartzentruber D.J., Kaufman H.L., Agarwala S.S., Tarhini A.A., Lowder J.N., Atkins M.B. (2014). High Dose Interleukin-2 (Aldesleukin)—Expert Consensus on Best Management Practices-2014. J. Immunother. Cancer.

[5] Mansh M. (2011). Ipilimumab and Cancer Immunotherapy: A New Hope for Advanced Stage Melanoma. Yale J. Biol. Med..

[6] Shu C.A., Rizvi N.A. (2016). Into the Clinic with Nivolumab and Pembrolizumab. Oncologist.

[7] Rotte A., D’Orazi G., Bhandaru M. (2018). Nobel Committee Honors Tumor Immunologists. J. Exp. Clin. Cancer Res..

[8] Huehls A.M., Coupet T.A., Sentman C.L. (2015). Bispecific T-Cell Engagers for Cancer Immunotherapy. Immunol. Cell Biol..

[9] Przepiorka D., Ko C.-W., Deisseroth A., Yancey C.L., Candau-Chacon R., Chiu H.-J., Gehrke B.J., Gomez-Broughton C., Kane R.C., Kirshner S. (2015). FDA Approval: Blinatumomab. Clin. Cancer Res..

[10] Rosenberg S.A., Spiess P., Lafreniere R. (1986). A New Approach to the Adoptive Immunotherapy of Cancer with Tumor-Infiltrating Lymphocytes. Science.

[11] Kuwana Y., Asakura Y., Utsunomiya N., Nakanishi M., Arata Y., Itoh S., Nagase F., Kurosawa Y. (1987). Expression of Chimeric Receptor Composed of Immunoglobulin-Derived V Regions and T-Cell Receptor-Derived C Regions. Biochem. Biophys. Res. Commun..

[12] Eshhar Z., Waks T., Gross G., Schindler D.G. (1993). Specific Activation and Targeting of Cytotoxic Lymphocytes through Chimeric Single Chains Consisting of Antibody-Binding Domains and the Gamma or Zeta Subunits of the Immunoglobulin and T-Cell Receptors. Proc. Natl. Acad. Sci. USA.

[13] Gross G., Waks T., Eshhar Z. (1989). Expression of Immunoglobulin-T-Cell Receptor Chimeric Molecules as Functional Receptors with Antibody-Type Specificity. Proc. Natl. Acad. Sci. USA.

[14] Gross G., Gorochov G., Waks T., Eshhar Z. (1989). Generation of Effector T Cells Expressing Chimeric T Cell Receptor with Antibody Type-Specificity. Transpl. Proc..

[15] Mitra A., Barua A., Huang L., Ganguly S., Feng Q., He B. (2023). From Bench to Bedside: The History and Progress of CAR T Cell Therapy. Front. Immunol..

[16] Kochenderfer J.N. (2024). FDA Approval of the First Cellular Therapy for a Solid (Non-Hematologic) Cancer. Mol. Ther..

[17] Liu Y.-T., Sun Z.-J. (2021). Turning Cold Tumors into Hot Tumors by Improving T-Cell Infiltration. Theranostics.

[18] Bonaventura P., Shekarian T., Alcazer V., Valladeau-Guilemond J., Valsesia-Wittmann S., Amigorena S., Caux C., Depil S. (2019). Cold Tumors: A Therapeutic Challenge for Immunotherapy. Front. Immunol..

[19] Dewhirst M.W., Secomb T.W. (2017). Transport of Drugs from Blood Vessels to Tumour Tissue. Nat. Rev. Cancer.

[20] Bartelink I.H., Jones E.F., Shahidi-Latham S.K., Lee P.R.E., Zheng Y., Vicini P., van ’t Veer L., Wolf D., Iagaru A., Kroetz D.L. (2019). Tumor Drug Penetration Measurements Could Be the Neglected Piece of the Personalized Cancer Treatment Puzzle. Clin. Pharmacol. Ther..

[21] Chen T., Wang M., Chen Y., Liu Y. (2024). Current Challenges and Therapeutic Advances of CAR-T Cell Therapy for Solid Tumors. Cancer Cell Int..

[22] Basak S., Das T.K., Ganguly S. (2026). A Review on Tumor-Targeting Liposomes: Fabrication, Mechanism and Applications. Biochem. Pharmacol..

[23] Zugasti I., Espinosa-Aroca L., Fidyt K., Mulens-Arias V., Diaz-Beya M., Juan M., Urbano-Ispizua Á., Esteve J., Velasco-Hernandez T., Menéndez P. (2025). CAR-T Cell Therapy for Cancer: Current Challenges and Future Directions. Signal Transduct. Target. Ther..

[24] Lonez C., Breman E. (2024). Allogeneic CAR-T Therapy Technologies: Has the Promise Been Met?. Cells.

[25] Diorio C., Teachey D.T., Grupp S.A. (2025). Allogeneic Chimeric Antigen Receptor Cell Therapies for Cancer: Progress Made and Remaining Roadblocks. Nat. Rev. Clin. Oncol..

[26] Li Y.-R., Zhu Y., Fang Y., Lyu Z., Yang L. (2025). Emerging Trends in Clinical Allogeneic CAR Cell Therapy. Med.

[27] Pardoll D.M. (2012). The Blockade of Immune Checkpoints in Cancer Immunotherapy. Nat. Rev. Cancer.

[28] Rotte A. (2019). Combination of CTLA-4 and PD-1 Blockers for Treatment of Cancer. J. Exp. Clin. Cancer Res..

[29] Smith-Garvin J.E., Koretzky G.A., Jordan M.S. (2009). T Cell Activation. Annu. Rev. Immunol..

[30] Brunet J.F., Denizot F., Luciani M.F., Roux-Dosseto M., Suzan M., Mattei M.G., Golstein P. (1987). A New Member of the Immunoglobulin Superfamily--CTLA-4. Nature.

[31] Krummel M.F., Allison J.P. (1995). CD28 and CTLA-4 Have Opposing Effects on the Response of T Cells to Stimulation. J. Exp. Med..

[32] Leach D.R., Krummel M.F., Allison J.P. (1996). Enhancement of Antitumor Immunity by CTLA-4 Blockade. Science.

[33] Fife B.T., Bluestone J.A. (2008). Control of Peripheral T-Cell Tolerance and Autoimmunity via the CTLA-4 and PD-1 Pathways. Immunol. Rev..

[34] Ishida Y., Agata Y., Shibahara K., Honjo T. (1992). Induced Expression of PD-1, a Novel Member of the Immunoglobulin Gene Superfamily, upon Programmed Cell Death. EMBO J..

[35] Iwai Y., Ishida M., Tanaka Y., Okazaki T., Honjo T., Minato N. (2002). Involvement of PD-L1 on Tumor Cells in the Escape from Host Immune System and Tumor Immunotherapy by PD-L1 Blockade. Proc. Natl. Acad. Sci. USA.

[36] Keir M.E., Butte M.J., Freeman G.J., Sharpe A.H. (2008). PD-1 and Its Ligands in Tolerance and Immunity. Annu. Rev. Immunol..

[37] Meng L., Wu H., Wu J., Ding P., He J., Sang M., Liu L. (2024). Mechanisms of Immune Checkpoint Inhibitors: Insights into the Regulation of Circular RNAS Involved in Cancer Hallmarks. Cell Death Dis..

[38] Sharma P., Goswami S., Raychaudhuri D., Siddiqui B.A., Singh P., Nagarajan A., Liu J., Subudhi S.K., Poon C., Gant K.L. (2023). Immune Checkpoint Therapy-Current Perspectives and Future Directions. Cell.

[39] Gong J., Chehrazi-Raffle A., Reddi S., Salgia R. (2018). Development of PD-1 and PD-L1 Inhibitors as a Form of Cancer Immunotherapy: A Comprehensive Review of Registration Trials and Future Considerations. J. Immunother. Cancer.

[40] Jiang Y., Chen M., Nie H., Yuan Y. (2019). PD-1 and PD-L1 in Cancer Immunotherapy: Clinical Implications and Future Considerations. Hum. Vaccines Immunother..

[41] Lemaire V., Shemesh C.S., Rotte A. (2021). Pharmacology-Based Ranking of Anti-Cancer Drugs to Guide Clinical Development of Cancer Immunotherapy Combinations. J. Exp. Clin. Cancer Res..

[42] Zhang Y., Yao Q., Pan Y., Fang X., Xu H., Zhao T., Zhu G., Jiang T., Li S., Cao H. (2023). Efficacy and Safety of PD-1/PD-L1 Checkpoint Inhibitors versus Anti-PD-1/PD-L1 Combined with Other Therapies for Tumors: A Systematic Review. Cancers.

[43] Cercek A., Lumish M., Sinopoli J., Weiss J., Shia J., Lamendola-Essel M., El Dika I.H., Segal N., Shcherba M., Sugarman R. (2022). PD-1 Blockade in Mismatch Repair-Deficient, Locally Advanced Rectal Cancer. N. Engl. J. Med..

[44] GSK Jemperli (Dostarlimab) Receives US FDA Breakthrough Therapy Designation for Locally Advanced dMMR/MSI-H Rectal Cancer. https://www.gsk.com/en-gb/media/press-releases/jemperli-dostarlimab-receives-us-fda-breakthrough-therapy-designation-for-locally-advanced-dmmrmsi-h-rectal-cancer/.

[45] So W.V., Dejardin D., Rossmann E., Charo J. (2023). Predictive Biomarkers for PD-1/PD-L1 Checkpoint Inhibitor Response in NSCLC: An Analysis of Clinical Trial and Real-World Data. J. Immunother. Cancer.

[46] Li H., Van Der Merwe P.A., Sivakumar S. (2022). Biomarkers of Response to PD-1 Pathway Blockade. Br. J. Cancer.

[47] Mariam A., Kamath S., Schveder K., McLeod H.L., Rotroff D.M. (2023). Biomarkers for Response to Anti-PD-1/Anti-PD-L1 Immune Checkpoint Inhibitors: A Large Meta-Analysis. Oncology.

[48] FDA List of Cleared or Approved Companion Diagnostic Devices (In Vitro and Imaging Tools). https://www.fda.gov/medical-devices/in-vitro-diagnostics/list-cleared-or-approved-companion-diagnostic-devices-in-vitro-and-imaging-tools.

[49] Zhang T., Forde P.M., Sullivan R.J., Sharon E., Barksdale E., Selig W., Ebbinghaus S., Fusaro G., Gunenc D., Battle D. (2023). Addressing Resistance to PD-1/PD-(L)1 Pathway Inhibition: Considerations for Combinatorial Clinical Trial Designs. J. Immunother. Cancer.

[50] Javed S.A., Najmi A., Ahsan W., Zoghebi K. (2024). Targeting PD-1/PD-L-1 Immune Checkpoint Inhibition for Cancer Immunotherapy: Success and Challenges. Front. Immunol..

[51] Sun J.-Y., Zhang D., Wu S., Xu M., Zhou X., Lu X.-J., Ji J. (2020). Resistance to PD-1/PD-L1 Blockade Cancer Immunotherapy: Mechanisms, Predictive Factors, and Future Perspectives. Biomark. Res..

[52] Chamoto K., Hatae R., Honjo T. (2020). Current Issues and Perspectives in PD-1 Blockade Cancer Immunotherapy. Int. J. Clin. Oncol..

[53] Garcia-Diaz A., Shin D.S., Moreno B.H., Saco J., Escuin-Ordinas H., Rodriguez G.A., Zaretsky J.M., Sun L., Hugo W., Wang X. (2017). Interferon Receptor Signaling Pathways Regulating PD-L1 and PD-L2 Expression. Cell Rep..

[54] Zhang H., Hu Y., Wu T., Chen Y., Yang B., Xie T. (2023). Clinical Characteristics and Novel Strategies of Immune Checkpoint Inhibitor Rechallenge Therapy for Non-Small Cell Lung Cancer: A Comprehensive Review. Front. Immunol..

[55] Liu S.-J., Yan L.-J., Wang H.-C., Ding Z.-N., Liu H., Zhang X., Pan G.-Q., Han C.-L., Tian B.-W., Yang X.-R. (2024). Safety, Efficacy, and Survival Outcomes of Immune Checkpoint Inhibitors Rechallenge in Patients with Cancer: A Systematic Review and Meta-Analysis. Oncologist.

[56] Haanen J., Ernstoff M., Wang Y., Menzies A., Puzanov I., Grivas P., Larkin J., Peters S., Thompson J., Obeid M. (2020). Rechallenge Patients with Immune Checkpoint Inhibitors Following Severe Immune-Related Adverse Events: Review of the Literature and Suggested Prophylactic Strategy. J. Immunother. Cancer.

[57] Hu H., Wang K., Jia R., Zeng Z.-X., Zhu M., Deng Y.-L., Xiong Z.-J., Tang J.-N., Xie H., Wang Y. (2023). Current Status in Rechallenge of Immunotherapy. Int. J. Biol. Sci..

[58] Da Silva I.P., Zimmer L., Blay J.-Y., Maio M., Larkin J., Grimm M.-O., Puri S., Butler M.O., Patel S., Thakkar P.K. (2025). Retreatment, Rechallenge, and Escalation with Subsequent Immune Checkpoint Inhibitor Therapies across Cancers after Initial Failure. ESMO Open.

[59] Feng J., Chen X., Wei J., Weng Y., Wang J., Wang T., Song Q., Min P. (2024). Safety and Efficacy of Immune Checkpoint Inhibitor Rechallenge in Advanced Non-Small Cell Lung Cancer: A Retrospective Study. Sci. Rep..

[60] Kothari M., Wanjari A., Acharya S., Karwa V., Chavhan R., Kumar S., Kadu A., Patil R. (2024). A Comprehensive Review of Monoclonal Antibodies in Modern Medicine: Tracing the Evolution of a Revolutionary Therapeutic Approach. Cureus.

[61] Zahavi D., Weiner L. (2020). Monoclonal Antibodies in Cancer Therapy. Antibodies.

[62] Sedykh S.E., Prinz V.V., Buneva V.N., Nevinsky G.A. (2018). Bispecific Antibodies: Design, Therapy, Perspectives. Drug Des. Dev. Ther..

[63] Li T., Niu M., Zhou J., Wu K., Yi M. (2024). The Enhanced Antitumor Activity of Bispecific Antibody Targeting PD-1/PD-L1 Signaling. Cell Commun. Signal.

[64] Shah V., McNatty A., Simpson L., Ofori H., Raheem F. (2023). Amivantamab-Vmjw: A Novel Treatment for Patients with NSCLC Harboring EGFR Exon 20 Insertion Mutation after Progression on Platinum-Based Chemotherapy. Biomedicines.

[65] Xue J., Ma Y., Zhao Y., Wang Y., Hong W., Huang Y., Yang Y., Fang W., Hong S., Zhang Y. (2025). Izalontamab (SI-B001), a Novel EGFRxHER3 Bispecific Antibody in Patients with Locally Advanced or Metastatic Epithelial Tumor: Results from First-in-Human Phase I/Ib Study. Clin. Cancer Res..

[66] Van Herpen C.M.L., Daste A., Arrazubi V., De Boer J.P., Rojas K.I., Clatot F., Fontana E., Harandi A., Hellyer J., Hollebecque A. (2025). Petosemtamab (MCLA-158) with Pembrolizumab as First-Line (1L) Treatment of PD-L1+ Recurrent/Metastatic (r/m) Head and Neck Squamous Cell Carcinoma (HNSCC): Phase 2 Trial. J. Clin. Oncol..

[67] Wang J., Lou H., Cai H.-B., Huang X., Li G., Wang L., Liu T., Liu W., Li B., Xia Y. (2022). A Study of AK104 (an Anti-PD1 and Anti-CTLA4 Bispecific Antibody) Combined with Standard Therapy for the First-Line Treatment of Persistent, Recurrent, or Metastatic Cervical Cancer (R/M CC). J. Clin. Oncol..

[68] Pang X., Huang Z., Zhong T., Zhang P., Wang Z.M., Xia M., Li B. (2023). Cadonilimab, a Tetravalent PD-1/CTLA-4 Bispecific Antibody with Trans-Binding and Enhanced Target Binding Avidity. mAbs.

[69] Voss M.H., Garmezy B., Kim S.H., Maroto Rey J.P., Mansinho A.B., Rodriguez-Vida A., Oliveira J., Van Dongen M., Rodríguez L.M., Negrier S. (2023). 1883MO MEDI5752 (Volrustomig), a Novel PD-1/CTLA-4 Bispecific Antibody, in the First-Line (1L) Treatment of 65 Patients (Pts) with Advanced Clear Cell Renal Cell Carcinoma (aRCC). Ann. Oncol..

[70] Zhao Y., Chen G., Li X., Wu J., Chang B., Hu S., Yang S., Xu T., Liu Y., Wang N. (2024). KN046, a Bispecific Antibody against PD-L1 and CTLA-4, plus Chemotherapy as First-Line Treatment for Metastatic NSCLC: A Multicenter Phase 2 Trial. Cell Rep. Med..

[71] Hack S.P., Zhu A.X., Wang Y. (2020). Augmenting Anticancer Immunity Through Combined Targeting of Angiogenic and PD-1/PD-L1 Pathways: Challenges and Opportunities. Front. Immunol..

[72] Zhou Y., Li J., Ying J. (2024). Anti-PD-1/L1 Antibody plus Anti-VEGF Antibody vs. plus VEGFR-Targeted TKI as First-Line Therapy for Unresectable Hepatocellular Carcinoma: A Network Meta-Analysis. Explor. Target. Antitumor Ther..

[73] Kudo M. (2020). Scientific Rationale for Combined Immunotherapy with PD-1/PD-L1 Antibodies and VEGF Inhibitors in Advanced Hepatocellular Carcinoma. Cancers.

[74] Yi M., Jiao D., Qin S., Chu Q., Wu K., Li A. (2019). Synergistic Effect of Immune Checkpoint Blockade and Anti-Angiogenesis in Cancer Treatment. Mol. Cancer.

[75] Fang W., Zhao Y., Luo Y., Yang R., Huang Y., He Z., Zhao H., Li M., Li K., HARMONi-A Study Investigators (2024). Ivonescimab Plus Chemotherapy in Non-Small Cell Lung Cancer with EGFR Variant: A Randomized Clinical Trial. JAMA.

[76] Xiong A., Wang L., Chen J., Wu L., Liu B., Yao J., Zhong H., Li J., Cheng Y., Sun Y. (2025). Ivonescimab versus Pembrolizumab for PD-L1-Positive Non-Small Cell Lung Cancer (HARMONi-2): A Randomised, Double-Blind, Phase 3 Study in China. Lancet.

[77] Wu L., Yao J., Sun Y., Wang R., Li X., Chen B., Chu Q., Bu Q., Fang Y., Zhao J. (2025). A Phase II Trial to Evaluate the Safety and Efficacy of SSGJ-707, a Bispecific Antibody Targeting PD-1 and VEGF, as a Monotherapy in Patients with Advanced NSCLC. J. Clin. Oncol..

[78] Wu L., Xu H., Sun Y., Ning F., Huang S., Huang D., Yu Y., Ye F., Lv D., Pei Z. (2025). 1328 SSGJ-707, a PD-1/VEGF Bispecific Antibody, Combined with Platinum-Based Chemotherapy in First-Line Treatment of Advanced Non-Small Cell Lung Cancer (NSCLC): Results from a Phase 2 Study. Proceedings of the Late Breaking Abstracts.

[79] Heymach J.V., Cho B.C., Sezer A., Karacin C., Lee Y.J., Çil T., Ozyilkan O., Arslan C., Lee G.-W., Basak Oven B. (2025). OA13.02 Global Phase 2 Randomized Trial of BNT327 (Pumitamig; PD-L1 × VEGF-A bsAb) + Chemotherapy for 1L ES-SCLC: Dose Optimization Analysis. J. Thorac. Oncol..

[80] Zhao Y., Chen G., Chen J., Zhuang L., Du Y., Yu Q., Zhuang W., Zhao Y., Zhou M., Zhang W. (2023). AK112, a Novel PD-1/VEGF Bispecific Antibody, in Combination with Chemotherapy in Patients with Advanced Non-Small Cell Lung Cancer (NSCLC): An Open-Label, Multicenter, Phase II Trial. eClinicalMedicine.

[81] Baldwin W.M., Valujskikh A., Fairchild R.L. (2019). The Neonatal Fc Receptor: Key to Homeostasic Control of IgG and IgG-Related Biopharmaceuticals. Am. J. Transplant..

[82] de Taeye S.W., Rispens T., Vidarsson G. (2019). The Ligands for Human IgG and Their Effector Functions. Antibodies.

[83] Lu L.L., Suscovich T.J., Fortune S.M., Alter G. (2018). Beyond Binding: Antibody Effector Functions in Infectious Diseases. Nat. Rev. Immunol..

[84] Wang M., Wang C., Deng J., Wang H., Sun C., Luo S., Hu Y. (2024). Bispecific Antibodies for Multiple Myeloma: Recent Advancements and Strategies for Increasing Their Efficacy. Front. Biosci..

[85] Fan G., Wang Z., Hao M., Li J. (2015). Bispecific Antibodies and Their Applications. J. Hematol. Oncol..

[86] Mohan N., Ayinde S., Peng H., Dutta S., Shen Y., Falkowski V.M., Biel T.G., Ju T., Wu W.J. (2024). Structural and Functional Characterization of IgG- and Non-IgG-Based T-Cell-Engaging Bispecific Antibodies. Front. Immunol..

[87] Dewaele L., Fernandes R.A. (2025). Bispecific T-Cell Engagers for the Recruitment of T Cells in Solid Tumors: A Literature Review. Immunother. Adv..

[88] Kantarjian H., Stein A., Gökbuget N., Fielding A.K., Schuh A.C., Ribera J.-M., Wei A., Dombret H., Foà R., Bassan R. (2017). Blinatumomab versus Chemotherapy for Advanced Acute Lymphoblastic Leukemia. N. Engl. J. Med..

[89] Mateos M.-V., Bahlis N., Perrot A., Nooka A., Lu J., Pawlyn C., Mina R., Caeiro G., Kentos A., Hungria V. (2025). Phase 3 Randomized Study of Teclistamab plus Daratumumab versus Investigator’s Choice of Daratumumab and Dexamethasone with Either Pomalidomide or Bortezomib (DPd/DVd) in Patients (Pts) with Relapsed Refractory Multiple Myeloma (RRMM): Results of Majestec-3. Blood.

[90] Albayrak G., Wan P.K.-T., Fisher K., Seymour L.W. (2025). T Cell Engagers: Expanding Horizons in Oncology and Beyond. Br. J. Cancer.

[91] Spinazzola A., Iannantuono G.M., Gulley J.L., Giudice E., Filetti M., Sganga S., Bianco F.L., Floudas C.S., Daniele G. (2025). Current Landscape of T-Cell Engagers in Early-Phase Clinical Development in Solid Cancers. Front. Immunol..

[92] Hassel J.C., Piperno-Neumann S., Rutkowski P., Baurain J.-F., Schlaak M., Butler M.O., Sullivan R.J., Dummer R., Kirkwood J.M., Orloff M. (2023). Three-Year Overall Survival with Tebentafusp in Metastatic Uveal Melanoma. N. Engl. J. Med..

[93] Mountzios G., Sun L., Cho B.C., Demirci U., Baka S., Gümüş M., Lugini A., Zhu B., Yu Y., Korantzis I. (2025). Tarlatamab in Small-Cell Lung Cancer after Platinum-Based Chemotherapy. N. Engl. J. Med..

[94] Shui L., Wu D., Yang K., Sun C., Li Q., Yin R. (2025). Bispecific Antibodies: Unleashing a New Era in Oncology Treatment. Mol. Cancer.

[95] Salih H.R., Jung G. (2019). The Challenges of Translation. EMBO Mol. Med..

[96] Yuraszeck T., Kasichayanula S., Benjamin J. (2017). Translation and Clinical Development of Bispecific T-cell Engaging Antibodies for Cancer Treatment. Clin. Pharmacol. Ther..

[97] Ball K., Dovedi S.J., Vajjah P., Phipps A. (2023). Strategies for Clinical Dose Optimization of T Cell-Engaging Therapies in Oncology. MAbs.

[98] Wang H., Zheng T., Xu D., Sun C., Huang D., Liu X. (2025). Targeting DLL3: Innovative Strategies for Tumor Treatment. Pharmaceutics.

[99] Lozada J.R., Elliott A., Evans M.G., Wacker J., Storey K.M., Egusa E.A., Zorko N.A., Kumar A., Crymes A., Heath E.I. (2025). Expression Patterns of DLL3 across Neuroendocrine and Non-Neuroendocrine Neoplasms Reveal Broad Opportunities for Therapeutic Targeting. Cancer Res. Commun..

[100] Kershaw M.H., Westwood J.A., Parker L.L., Wang G., Eshhar Z., Mavroukakis S.A., White D.E., Wunderlich J.R., Canevari S., Rogers-Freezer L. (2006). A Phase I Study on Adoptive Immunotherapy Using Gene-Modified T Cells for Ovarian Cancer. Clin. Cancer Res..

[101] Lamers C.H.J., Sleijfer S., Vulto A.G., Kruit W.H.J., Kliffen M., Debets R., Gratama J.W., Stoter G., Oosterwijk E. (2006). Treatment of Metastatic Renal Cell Carcinoma with Autologous T-Lymphocytes Genetically Retargeted against Carbonic Anhydrase IX: First Clinical Experience. J. Clin. Oncol..

[102] Till B.G., Jensen M.C., Wang J., Chen E.Y., Wood B.L., Greisman H.A., Qian X., James S.E., Raubitschek A., Forman S.J. (2008). Adoptive Immunotherapy for Indolent Non-Hodgkin Lymphoma and Mantle Cell Lymphoma Using Genetically Modified Autologous CD20-Specific T Cells. Blood.

[103] Savoldo B., Ramos C.A., Liu E., Mims M.P., Keating M.J., Carrum G., Kamble R.T., Bollard C.M., Gee A.P., Mei Z. (2011). CD28 Costimulation Improves Expansion and Persistence of Chimeric Antigen Receptor-Modified T Cells in Lymphoma Patients. J. Clin. Investig..

[104] Chmielewski M., Hombach A.A., Abken H. (2014). Of CAR s and TRUCK s: Chimeric Antigen Receptor ( CAR ) T Cells Engineered with an Inducible Cytokine to Modulate the Tumor Stroma. Immunol. Rev..

[105] Chmielewski M., Abken H. (2015). TRUCKs: The Fourth Generation of CARs. Expert Opin. Biol. Ther..

[106] Anupindi K., Malachowski J., Hodson I., Zhu D., June C.H., Levine B.L. (2025). The next Innovations in Chimeric Antigen Receptor T Cell Immunotherapies for Cancer. Cytotherapy.

[107] Asmamaw Dejenie T., Tiruneh G M., Dessie Terefe G., Tadele Admasu F., Wale Tesega W., Chekol Abebe E. (2022). Current Updates on Generations, Approvals, and Clinical Trials of CAR T-Cell Therapy. Hum. Vaccines Immunother..

[108] Dagar G., Gupta A., Masoodi T., Nisar S., Merhi M., Hashem S., Chauhan R., Dagar M., Mirza S., Bagga P. (2023). Harnessing the Potential of CAR-T Cell Therapy: Progress, Challenges, and Future Directions in Hematological and Solid Tumor Treatments. J. Transl. Med..

[109] Kagoya Y., Tanaka S., Guo T., Anczurowski M., Wang C.-H., Saso K., Butler M.O., Minden M.D., Hirano N. (2018). A Novel Chimeric Antigen Receptor Containing a JAK-STAT Signaling Domain Mediates Superior Antitumor Effects. Nat. Med..

[110] Yuti P., Sawasdee N., Natungnuy K., Rujirachaivej P., Luangwattananun P., Sujjitjoon J., Yenchitsomanus P.-T. (2023). Enhanced Antitumor Efficacy, Proliferative Capacity, and Alleviation of T Cell Exhaustion by Fifth-Generation Chimeric Antigen Receptor T Cells Targeting B Cell Maturation Antigen in Multiple Myeloma. Biomed. Pharmacother..

[111] Zhang C., Liu J., Zhong J.F., Zhang X. (2017). Engineering CAR-T Cells. Biomark. Res..

[112] Huang R., Li X., He Y., Zhu W., Gao L., Liu Y., Gao L., Wen Q., Zhong J.F., Zhang C. (2020). Recent Advances in CAR-T Cell Engineering. J. Hematol. Oncol..

[113] Maus M.V., June C.H. (2013). Zoom Zoom: Racing CARs for Multiple Myeloma. Clin. Cancer Res..

[114] Larson R.C., Maus M.V. (2021). Recent Advances and Discoveries in the Mechanisms and Functions of CAR T Cells. Nat. Rev. Cancer.

[115] Rotte A., Frigault M.J., Ansari A., Gliner B., Heery C., Shah B. (2022). Dose-Response Correlation for CAR-T Cells: A Systematic Review of Clinical Studies. J. Immunother. Cancer.

[116] Fesnak A.D., June C.H., Levine B.L. (2016). Engineered T Cells: The Promise and Challenges of Cancer Immunotherapy. Nat. Rev. Cancer.

[117] Firor A.E., Jares A., Ma Y. (2015). From Humble Beginnings to Success in the Clinic: Chimeric Antigen Receptor-Modified T-Cells and Implications for Immunotherapy. Exp. Biol. Med..

[118] Grigor E.J.M., Fergusson D., Kekre N., Montroy J., Atkins H., Seftel M.D., Daugaard M., Presseau J., Thavorn K., Hutton B. (2019). Risks and Benefits of Chimeric Antigen Receptor T-Cell (CAR-T) Therapy in Cancer: A Systematic Review and Meta-Analysis. Transfus. Med. Rev..

[119] Ivica N.A., Young C.M. (2021). Tracking the CAR-T Revolution: Analysis of Clinical Trials of CAR-T and TCR-T Therapies for the Treatment of Cancer (1997–2020). Healthcare.

[120] Mohanty R., Chowdhury C.R., Arega S., Sen P., Ganguly P., Ganguly N. (2019). CAR T Cell Therapy: A New Era for Cancer Treatment. Oncol. Rep..

[121] Sun D., Shi X., Li S., Wang X., Yang X., Wan M. (2024). CAR-T Cell Therapy: A Breakthrough in Traditional Cancer Treatment Strategies. Mol. Med. Rep..

[122] Verdun N., Marks P. (2024). Secondary Cancers after Chimeric Antigen Receptor T-Cell Therapy. N. Engl. J. Med..

[123] Aleman A., Van Oekelen O., Melnekoff D.T., Grossman L., Mouhieddine T.H., Kurowski A., Odak I., Reci S., Desai S., Meledathu S. (2025). Targeted Therapy of CAR+ T-Cell Lymphoma after Anti-BCMA CAR T-Cell Therapy. N. Engl. J. Med..

[124] Darren Incorvaia “The Only Thing That Saves Us Is Data”: Allogeneic CAR-T Biotechs Fight for Relevance as Industry Moves On. https://www.fiercebiotech.com/biotech/only-thing-saves-us-data-allogeneic-car-t-biotechs-fight-relevance-industry-moves.

[125] Pinto E., Lione L., Compagnone M., Paccagnella M., Salvatori E., Greco M., Frezza V., Marra E., Aurisicchio L., Roscilli G. (2025). From Ex Vivo to in Vivo Chimeric Antigen T Cells Manufacturing: New Horizons for CAR T-Cell Based Therapy. J. Transl. Med..

[126] Escobar G., Berger T.R., Maus M.V. (2025). CAR-T Cells in Solid Tumors: Challenges and Breakthroughs. Cell Rep. Med..

[127] Rojas-Quintero J., Díaz M.P., Palmar J., Galan-Freyle N.J., Morillo V., Escalona D., González-Torres H.J., Torres W., Navarro-Quiroz E., Rivera-Porras D. (2024). Car T Cells in Solid Tumors: Overcoming Obstacles. Int. J. Mol. Sci..

[128] Qi C., Liu C., Gong J., Liu D., Wang X., Zhang P., Qin Y., Ge S., Zhang M., Peng Z. (2024). Claudin18.2-Specific CAR T Cells in Gastrointestinal Cancers: Phase 1 Trial Final Results. Nat. Med..

[129] Qi C., Liu C., Peng Z., Zhang Y., Wei J., Qiu W., Zhang X., Pan H., Niu Z., Qiu M. (2025). Claudin-18 Isoform 2-Specific CAR T-Cell Therapy (Satri-Cel) versus Treatment of Physician’s Choice for Previously Treated Advanced Gastric or Gastro-Oesophageal Junction Cancer (CT041-ST-01): A Randomised, Open-Label, Phase 2 Trial. Lancet.

[130] CARsgenTherapeutics CARsgen Announces CT041 CAR T-Cell Product Candidate Granted RMAT Designation by the FDA. https://www.carsgen.com/en/news/carsgen-announces-ct041-car-t-cell-product-candidate-granted-rmat-designation-by-the-fda/.

[131] Bui T.A., Mei H., Sang R., Ortega D.G., Deng W. (2024). Advancements and Challenges in Developing in Vivo CAR T Cell Therapies for Cancer Treatment. eBioMedicine.

[132] Bushman F.D. (2020). Retroviral Insertional Mutagenesis in Humans: Evidence for Four Genetic Mechanisms Promoting Expansion of Cell Clones. Mol. Ther..

[133] Zhao Y., Deng J., Rao S., Guo S., Shen J., Du F., Wu X., Chen Y., Li M., Chen M. (2022). Tumor Infiltrating Lymphocyte (TIL) Therapy for Solid Tumor Treatment: Progressions and Challenges. Cancers.

[134] Matsueda S., Chen L., Li H., Yao H., Yu F. (2024). Recent Clinical Researches and Technological Development in TIL Therapy. Cancer Immunol. Immunother..

[135] Rosenberg S.A., Packard B.S., Aebersold P.M., Solomon D., Topalian S.L., Toy S.T., Simon P., Lotze M.T., Yang J.C., Seipp C.A. (1988). Use of Tumor-Infiltrating Lymphocytes and Interleukin-2 in the Immunotherapy of Patients with Metastatic Melanoma. A Preliminary Report. N. Engl. J. Med..

[136] Paijens S.T., Vledder A., de Bruyn M., Nijman H.W. (2021). Tumor-Infiltrating Lymphocytes in the Immunotherapy Era. Cell. Mol. Immunol..

[137] Wang S., Sun J., Chen K., Ma P., Lei Q., Xing S., Cao Z., Sun S., Yu Z., Liu Y. (2021). Perspectives of Tumor-Infiltrating Lymphocyte Treatment in Solid Tumors. BMC Med..

[138] Zhu C., Zhao Y., He J., Zhao H., Ni L., Cheng X., Chen Y., Mu L., Zhou X., Shi Q. (2023). TIL-Derived CAR T Cells Improve Immune Cell Infiltration and Survival in the Treatment of CD19-Humanized Mouse Colorectal Cancer. Cancers.

[139] Chen R., Johnson J., Rezazadeh A., Dudek A.Z. (2025). Tumour-Infiltrating Lymphocyte Therapy Landscape: Prospects and Challenges. BMJ Oncol..

[140] (2013). Decade-Long Survival Possible after Ipilimumab. Cancer Discov..

[141] Li J., Gu J. (2019). Efficacy and Safety of Ipilimumab for Treating Advanced Melanoma: A Systematic Review and Meta-analysis. J. Clin. Pharm. Ther..

[142] O’Byrne K., Popoff E., Badin F., Lee A., Yuan Y., Lozano-Ortega G., Eccles L.J., Varol N., Waser N., Penrod J.R. (2023). Long-Term Comparative Efficacy and Safety of Nivolumab plus Ipilimumab Relative to Other First-Line Therapies for Advanced Non-Small-Cell Lung Cancer: A Systematic Literature Review and Network Meta-Analysis. Lung Cancer.

[143] Wang D.Y., Salem J.-E., Cohen J.V., Chandra S., Menzer C., Ye F., Zhao S., Das S., Beckermann K.E., Ha L. (2018). Fatal Toxic Effects Associated with Immune Checkpoint Inhibitors: A Systematic Review and Meta-Analysis. JAMA Oncol..

[144] Sun L., Zhang L., Yu J., Zhang Y., Pang X., Ma C., Shen M., Ruan S., Wasan H.S., Qiu S. (2020). Clinical Efficacy and Safety of Anti-PD-1/PD-L1 Inhibitors for the Treatment of Advanced or Metastatic Cancer: A Systematic Review and Meta-Analysis. Sci. Rep..

[145] Wang Y., Zhou S., Yang F., Qi X., Wang X., Guan X., Shen C., Duma N., Vera Aguilera J., Chintakuntlawar A. (2019). Treatment-Related Adverse Events of PD-1 and PD-L1 Inhibitors in Clinical Trials: A Systematic Review and Meta-Analysis. JAMA Oncol..

[146] Zhou X., Yao Z., Bai H., Duan J., Wang Z., Wang X., Zhang X., Xu J., Fei K., Zhang Z. (2021). Treatment-Related Adverse Events of PD-1 and PD-L1 Inhibitor-Based Combination Therapies in Clinical Trials: A Systematic Review and Meta-Analysis. Lancet Oncol..

[147] Gobbini E., Charles J., Toffart A.-C., Leccia M.-T., Moro-Sibilot D., Levra M.G. (2020). Literature Meta-Analysis about the Efficacy of Re-Challenge with PD-1 and PD-L1 Inhibitors in Cancer Patients. Bull. Cancer.

[148] Kreft S., Bosetti T., Lee R., Lorigan P. (2025). Selecting First-Line Immunotherapy in Advanced Melanoma: Current Evidence on Efficacy across Diverse Patient Populations. EJC Ski. Cancer.

[149] Zhu J., Wu Q., Yang Z., Wu C., Zhou J., Li S., Wu J., Jiang J. (2025). Safety Profile of Nivolumab-Relatlimab in Cancer Patients: A Living Pharmacovigilance Study of Clinical Trials and Postmarketing Data (Version 1.0). Eur. J. Pharmacol..

[150] Davar D., Anderson A.C., Diaz-Padilla I. (2025). Therapeutic Potential of Targeting LAG-3 in Cancer. J. Immunother. Cancer.

[151] Nejati N., Robat-Jazi B., Saleh K., Dashti M., Zand A., Lorestani P., Karami S., Bahri Najafi M., Rastgou P., Rahimikia F. (2025). The Safety and Efficacy of Anti-LAG-3 for Patients with Melanoma: A Systematic Review and Meta-Analysis Study. Anticancer Agents Med. Chem..

[152] Subklewe M. (2021). BiTEs Better than CAR T Cells. Blood Adv..

[153] Zhai Y., Hong J., Wang J., Jiang Y., Wu W., Lv Y., Guo J., Tian L., Sun H., Li Y. (2024). Comparison of Blinatumomab and CAR T-Cell Therapy in Relapsed/Refractory Acute Lymphoblastic Leukemia: A Systematic Review and Meta-Analysis. Expert Rev. Hematol..

[154] Guo H.-P., Liu Y., Kang L., Liu C., Qin W.-W. (2024). Efficacy and Safety of Blinatumomab for the Treatment of Patients Relapsing after Allogeneic Hematopoietic Cell Transplantation: A Systemic Review and Meta-Analysis. Hematology.

[155] Liu H., Xi R., Mao D., Zhao X., Wu T. (2023). Efficacy and Safety of Blinatumomab for the Treatment of Relapsed/Refractory Acute Lymphoblastic Leukemia: A Systemic Review and Meta-Analysis. Clin. Lymphoma Myeloma Leuk..

[156] Reynolds G., Cliff E.R.S., Mohyuddin G.R., Popat R., Midha S., Liet Hing M.N., Harrison S.J., Kesselheim A.S., Teh B.W. (2023). Infections Following Bispecific Antibodies in Myeloma: A Systematic Review and Meta-Analysis. Blood Adv..

[157] Liang X., Wang Y., Luo B., Lin B., Lu W., Tian S., Liu D., Wang L. (2024). Comparison of CAR T-Cell and Bispecific Antibody as Third-Line or Later-Line Treatments for Multiple Myeloma: A Meta-Analysis. J. Immunother. Cancer.

[158] Shen J., Zhang J., Zhu Z., Ma H., Li X., Zhang J., Zhou F., Tian H., Liu J. (2025). CD20×CD3 Bispecific Antibody Achieved Significant Efficacy in Patients with Large B-Cell Lymphoma Relapsing after or Refractory to CAR-T Therapy: A Systematic Review and Meta-Analysis. Front. Oncol..

[159] Techaapornkun P., Rojpalakorn W., Mejun N., Khaniya A., Thammahong A., Thu M.S., Hirankarn N., Pitakkitnukun P. (2025). Comparative Efficacy and Safety of BCMA-Targeted CAR T Cells and BiTEs in Relapsed/Refractory Multiple Myeloma: A Meta-Analysis of Interventional and Real-World Studies. Ann. Hematol..

[160] Bayly-McCredie E., Treisman M., Fiorenza S. (2024). Safety and Efficacy of Bispecific Antibodies in Adults with Large B-Cell Lymphomas: A Systematic Review of Clinical Trial Data. Int. J. Mol. Sci..

[161] Sands J.M., Champiat S., Hummel H.-D., Paulson K.G., Borghaei H., Alvarez J.B., Carbone D.P., Carlisle J.W., Choudhury N.J., Clarke J.M. (2025). Practical Management of Adverse Events in Patients Receiving Tarlatamab, a Delta-like Ligand 3-Targeted Bispecific T-Cell Engager Immunotherapy, for Previously Treated Small Cell Lung Cancer. Cancer.

[162] Dowlati A., Hummel H.-D., Champiat S., Olmedo M.E., Boyer M., He K., Steeghs N., Izumi H., Johnson M.L., Yoshida T. (2024). Sustained Clinical Benefit and Intracranial Activity of Tarlatamab in Previously Treated Small Cell Lung Cancer: DeLLphi-300 Trial Update. J. Clin. Oncol..

[163] Nathan P., Hassel J.C., Rutkowski P., Baurain J.-F., Butler M.O., Schlaak M., Sullivan R.J., Ochsenreither S., Dummer R., Kirkwood J.M. (2021). Overall Survival Benefit with Tebentafusp in Metastatic Uveal Melanoma. N. Engl. J. Med..

[164] Dian Y., Liu Y., Zeng F., Sun Y., Deng G. (2024). Efficacy and Safety of Tebentafusp in Patients with Metastatic Uveal Melanoma: A Systematic Review and Meta-Analysis. Hum. Vaccines Immunother..

[165] Saldanha E.F., Noronha M.M., Reis P.C.A., Passos P.R.C., Filho V.O.C., Cappellaro A.P., Almeida L.F.C., Maselli-Shoueri J.H., Lopes C.D.H., Leite L.F. (2025). Tebentafusp in Metastatic Uveal Melanoma: A Meta-Analysis. Target. Oncol..

[166] Petzold A., Steeb T., Wessely A., Koch E.A.T., Vera J., Berking C., Heppt M.V. (2023). Is Tebentafusp Superior to Combined Immune Checkpoint Blockade and Other Systemic Treatments in Metastatic Uveal Melanoma? A Comparative Efficacy Analysis with Population Adjustment. Cancer Treat. Rev..

[167] Zavaleta-Monestel E., García-Montero J., Arguedas-Chacón S., Quesada-Villaseñor R., Barrantes-López M., Arroyo-Solís R., Zuñiga-Orlich C.E. (2024). Amivantamab: A Novel Advance in the Treatment of Non-Small Cell Lung Cancer. Cureus.

[168] Papassotiriou I., Kapogiannatos A., Makatsoris C., Bakogeorgou S., Mantogiannakou I., Roussou E., Souras G., Liakas D., Sergentanis T.N., Gavriatopoulou M. (2024). Efficacy and Safety of Amivantamab in Advanced or Metastatic EGFR-Mutant Non-Small Cell Lung Cancer: A Systematic Review. J. Clin. Med..

[169] Florez N., LeBoeuf N.R., Rotow J., Marks J.A., Sabari J.K., Arrieta O., Baldotto C., Gosain R., Zawisza D., McDonald S. (2025). Mitigation and Management of Adverse Events Associated with Amivantamab Therapy. Oncologist.

[170] Sun R., Ning Z., Qin H., Zhang W., Teng Y., Jin C., Liu J., Wang A. (2024). A Real-World Pharmacovigilance Study of Amivantamab-Related Cardiovascular Adverse Events Based on the FDA Adverse Event Reporting System (FAERS) Database. Sci. Rep..

[171] Anagnostou T., Riaz I.B., Hashmi S.K., Murad M.H., Kenderian S.S. (2020). Anti-CD19 Chimeric Antigen Receptor T-Cell Therapy in Acute Lymphocytic Leukaemia: A Systematic Review and Meta-Analysis. Lancet Haematol..

[172] Montagna E., de Campos N.S.P., Porto V.A., da Silva G.C.P., Suarez E.R. (2024). CD19 CAR T Cells for B Cell Malignancies: A Systematic Review and Meta-Analysis Focused on Clinical Impacts of CAR Structural Domains, Manufacturing Conditions, Cellular Product, Doses, Patient’s Age, and Tumor Types. BMC Cancer.

[173] Willyanto S.E., Alimsjah Y.A., Tanjaya K., Tuekprakhon A., Pawestri A.R. (2024). Comprehensive Analysis of the Efficacy and Safety of CAR T-Cell Therapy in Patients with Relapsed or Refractory B-Cell Acute Lymphoblastic Leukaemia: A Systematic Review and Meta-Analysis. Ann. Med..

[174] Xia Y., Zhang J., Li J., Zhang L., Li J., Fan L., Chen L. (2022). Cytopenias Following Anti-CD19 Chimeric Antigen Receptor (CAR) T Cell Therapy: A Systematic Analysis for Contributing Factors. Ann. Med..

[175] Aamir S., Anwar M.Y., Khalid F., Khan S.I., Ali M.A., Khattak Z.E. (2021). Systematic Review and Meta-Analysis of CD19-Specific CAR-T Cell Therapy in Relapsed/Refractory Acute Lymphoblastic Leukemia in the Pediatric and Young Adult Population: Safety and Efficacy Outcomes. Clin. Lymphoma Myeloma Leuk..

[176] Ng L.C.K., Lee X.H., Tan Y.C., Wong K.L., Chow J.C.Y., Ling V.W.T., Thong E.W.S., Chan E.H.L., Chan W.L., Samuel M. (2025). Systematic Review and Meta-Analysis: CAR-T vs Bispecific Antibody as Third or Later-Line Therapy for Follicular Lymphoma. Blood Cancer J..

[177] Roex G., Timmers M., Wouters K., Campillo-Davo D., Flumens D., Schroyens W., Chu Y., Berneman Z.N., Lion E., Luo F. (2020). Safety and Clinical Efficacy of BCMA CAR-T-Cell Therapy in Multiple Myeloma. J. Hematol. Oncol..

[178] Akhtar O.S., Sheeba B.A., Azad F., Alessi L., Hansen D., Alsina M., Baz R., Shain K., Grajales Cruz A., Castaneda Puglianini O. (2024). Safety and Efficacy of Anti-BCMA CAR-T Cell Therapy in Older Adults with Multiple Myeloma: A Systematic Review and Meta-Analysis. J. Geriatr. Oncol..

[179] Yang Q., Li X., Zhang F., Yang Q., Zhou W., Liu J. (2021). Efficacy and Safety of CAR-T Therapy for Relapse or Refractory Multiple Myeloma: A Systematic Review and Meta-Analysis. Int. J. Med. Sci..

[180] Zhang L., Shen X., Yu W., Li J., Zhang J., Zhang R., Li J., Chen L. (2021). Comprehensive Meta-Analysis of Anti-BCMA Chimeric Antigen Receptor T-Cell Therapy in Relapsed or Refractory Multiple Myeloma. Ann. Med..

[181] Chesney J., Lewis K.D., Kluger H., Hamid O., Whitman E., Thomas S., Wermke M., Cusnir M., Domingo-Musibay E., Phan G.Q. (2022). Efficacy and Safety of Lifileucel, a One-Time Autologous Tumor-Infiltrating Lymphocyte (TIL) Cell Therapy, in Patients with Advanced Melanoma after Progression on Immune Checkpoint Inhibitors and Targeted Therapies: Pooled Analysis of Consecutive Cohorts of the C-144-01 Study. J. Immunother. Cancer.

[182] Hu L., Fan C., Bross P., Das A., Cho E.S., Knudson K.M., Tegenge M., Gao Q., Brewer J.R., Theoret M.R. (2025). FDA Approval Summary: Lifileucel for Unresectable or Metastatic Melanoma Previously Treated with an Anti-PD-1-Based Immunotherapy. Clin. Cancer Res..

[183] Frey C., Etminan M. (2024). Adverse Events of PD-1, PD-L1, CTLA-4, and LAG-3 Immune Checkpoint Inhibitors: An Analysis of the FDA Adverse Events Database. Antibodies.

[184] Karimi A., Alilou S., Mirzaei H.R. (2021). Adverse Events Following Administration of Anti-CTLA4 Antibody Ipilimumab. Front. Oncol..

[185] Yan T., Long M., Liu C., Zhang J., Wei X., Li F., Liao D. (2025). Immune-Related Adverse Events with PD-1/PD-L1 Inhibitors: Insights from a Real-World Cohort of 2523 Patients. Front. Pharmacol..

[186] Boucheron T., Chiche L., Penaranda G., Souquet M., Pegliasco H., Deturmeny J., Brunel V., Barrière N., Arbault-Bitton C., Coquet E. (2025). Risk of Serious Immune-Related Adverse Events with Various PD1 and PD-L1 Inhibitors: A Single-Institution, Real-Life, Comparative Study. Ther. Clin. Risk Manag..

[187] Adkins S. (2019). CAR T-Cell Therapy: Adverse Events and Management. J. Adv. Pract. Oncol..

[188] Ludwig H., Terpos E., Van De Donk N., Mateos M.-V., Moreau P., Dimopoulos M.-A., Delforge M., Rodriguez-Otero P., San-Miguel J., Yong K. (2023). Prevention and Management of Adverse Events during Treatment with Bispecific Antibodies and CAR T Cells in Multiple Myeloma: A Consensus Report of the European Myeloma Network. Lancet Oncol..

[189] Dimitrov K., Merkle F., Dimitrov M., Merkle S., Hoover A., Bachanova V. (2026). Major Adverse Events with Chimeric Antigen Receptor T-Cell Therapy: Presentation, Diagnosis, and Resuscitation. Ann. Emerg. Med..

[190] Bonifant C.L., Jackson H.J., Brentjens R.J., Curran K.J. (2016). Toxicity and Management in CAR T-Cell Therapy. Mol. Ther. Oncolytics.

[191] Santomasso B.D., Nastoupil L.J., Adkins S., Lacchetti C., Schneider B.J., Anadkat M., Atkins M.B., Brassil K.J., Caterino J.M., Chau I. (2021). Management of Immune-Related Adverse Events in Patients Treated with Chimeric Antigen Receptor T-Cell Therapy: ASCO Guideline. J. Clin. Oncol..

[192] Betof Warner A., Hamid O., Komanduri K., Amaria R., Butler M.O., Haanen J., Nikiforow S., Puzanov I., Sarnaik A., Bishop M.R. (2024). Expert Consensus Guidelines on Management and Best Practices for Tumor-Infiltrating Lymphocyte Cell Therapy. J. Immunother. Cancer.

