# Supplementary material for: Targeting T-Cells for Cancer Treatment: Current Clinical Strategies and Challenges
Source: Biomedicines. 2026 Mar 13;14(3):654. doi: 10.3390/biomedicines14030654 (PMC13024664; doi:10.3390/biomedicines14030654)
Supplement: Supplementary file 1 [file biomedicines-14-00654-s001.zip › biomedicines-4136322-supplementary.pdf]

Supplementary Table S1. Efficacy outcomes reported for pembrolizumab (Pembro; PD-1 blocker)*

Cancer type	Study details	Comparator	Efficacy outcomes (Median reported in months; parentheses include 95% CI)
Melanoma	Open label, multicenter; KEYNOTE-006<br>Cohort 1: Pembro 10 mg/kg Q2W (n=278)<br>Cohort 2: Pembro 10 mg/kg Q3W (n=277)<br>Cohort 3: Ipi 3 mg/kg Q3W for 4 doses (n=256)	Ipilimumab	Q2W cohort<br><u>OS</u><br>Median: Not reported<br>HR: 0.69 (0.56-0.90)<br>ORR: 33% (27-39)<br><u>PFS</u><br>Median: 5.5 (3.4, 6.9)<br>HR: 0.58 (0.47-0.72)<br><br>Q3W cohort<br><u>OS</u><br>Median: Not reported<br>HR: 0.63 (0.47-0.83)<br><u>PFS</u><br>Median: 4.1 (2.9, 6.9)<br>HR: 0.58 (0.46-0.72)<br>ORR: 34% (28-40)
Non-small cell lung cancer	Double-blind, multicenter; KEYNOTE-189<br>Cohort 1: Pembro 200 mg Q3W, pemetrexed 500 mg/m2 and investigator-choice cisplatin 75 mg/m2 or carboplatin AUC 5 mg/mL/min<br>Cohort 2: Placebo; pemetrexed 500 mg/m2 and investigator-choice cisplatin 75 mg/m2 or carboplatin AUC 5 mg/mL/min	Placebo	<u>OS</u><br>Median: Not reached<br>HR: 0.49 (0.38, 0.64)<br><u>PFS</u><br>Median: 8.8 (7.6, 9.2)<br>HR: 0.52 (0.43, 0.64)<br>ORR: 48% (43, 53)
Non-small cell lung cancer	Open label, multicenter; KEYNOTE-042<br>Cohort 1: Pembro 200 mg Q3W<br>Cohort 2: Investigator-choice chemotherapy	Chemotherapy	Cohort TPS≥50%<br><u>OS</u><br>Median: 20.0 (15.4, 24.9)<br>HR: 0.69 (0.56, 0.85)<br><u>PFS</u><br>Median: 6.9 (5.9, 9.0)<br>HR: 0.82 (0.68, 0.99)<br>ORR: 39% (33.9, 45.3)
Malignant pleural mesothelioma	Open label, multicenter; KEYNOTE-483<br>Cohort 1: Pembro 200 mg Q3W, pemetrexed 500 mg/m2 and cisplatin 75 mg/m2 or carboplatin AUC 5 mg/mL/min IV<br>Cohort 2: Pemetrexed 500 mg/m2 and cisplatin 75 mg/m2 or carboplatin AUC 5 mg/mL/min IV	Chemotherapy	<u>OS</u><br>Median: 17.3 (14.4, 21.3)<br>HR: 0.79 (0.64, 0.98)<br><u>PFS</u><br>Median: 7.1 (6.9, 8.1)<br>HR: 0.80 (0.65, 0.99)<br>ORR: 52% (45.5, 59.0)

Head and neck squamous cell carcinoma	Open label, multicenter; KEYNOTE-048<br>Cohort 1: Pembro 200 mg Q3W<br>Cohort 2: Pembro 200 mg Q3W, carboplatin AUC 5 mg/mL/min IV or cisplatin 100 mg/m2 IV and FU 1000 mg/m2/day<br>Cohort 3: Cetuximab initial dose and carboplatin AUC 5 mg/mL/min IV or cisplatin 100 mg/m2 IV and FU 1000 mg/m2/day	Pembro plus chemotherapy vs Chemotherapy	OS<br>Median: 13.0 (10.9, 14.7)<br>HR: 0.77 (0.63, 0.93)<br>PFS<br>Median: 4.9 (4.7, 6.0)<br>HR: 0.92 (0.77, 1.10)<br>ORR: 36% (30.0, 41.5)
Urothelial cancer	Open label, multicenter, KEYNOTE-A39<br>Cohort 1: Pembro 200 mg Q3W and enfortumab vedotin 1.25 mg/kg<br>Cohort 2: Gemcitabine 1000 mg/m2 and cisplatin 70 mg/m2 or carboplatin (AUC = 4.5 or 5)	Chemotherapy	OS<br>Median: 31.5 (25.4, NR)<br>HR: 0.47 (0.38, 0.58)<br>PFS<br>Median: 12.5 (10.4, 16.6)<br>HR: 0.45 (0.38, 0.54)<br>ORR: 68% (68, 72)
Microsatellite Instability-High or Mismatch Repair deficient cancer	3 multicenter, non-randomized, open-label, multi-cohort trials: KEYNOTE-164, KEYNOTE-158, and KEYNOTE-051<br>Pembro 200 mg Q3W	Single arm	ORR: 33% (29, 38)<br>Duration of response: 63.2 months (1.9+-63.9+)
Microsatellite Instability-High or Mismatch Repair deficient colorectal cancer	Multicenter, open-label; KEYNOTE-177<br>Cohort 1: Pembro 200 mg Q3W<br>Cohort 2: Investigator choice chemotherapy	Chemotherapy	PFS<br>Median: 16.5 (5.4, 32.4)<br>HR: 0.60 (0.45, 0.80)<br>OS<br>Median: NR (49.2, NR)<br>HR: 0.74 (0.53, 1.03)<br>ORR: 44% (36, 52)
Gastric cancer	Multicenter, double-blind; KEYNOTE-811<br>Cohort 1: Pembro 200 mg Q3W, trastuzumab 6 mg/kg followed by investigator-choice chemotherapy<br>Cohort 2: placebo, trastuzumab 6 mg/kb followed by investigator-choice chemotherapy	Placebo combination	OS<br>Median: 20.1 (17.9, 22.9)<br>HR: 0.79 (0.66, 0.95)<br>PFS<br>Median: 10.9 (8.5, 12.5)<br>HR: 0.72 (0.60, 0.87)<br>ORR: 73% (68, 78)
Esophageal cancer	Multicenter, placebo controlled, KEYNOTE-590<br>Cohort 1: Pembro 200 mg Q3W, cisplatin 80 mg/m2 and FU 800 mg/m2<br>Cohort 2: Placebo, cisplatin 80 mg/m2 and FU 800 mg/m2	Placebo combination	Squamous cell histology CPS≥1 cohort<br>OS<br>Median: 12.7 (10.5, 14.4)<br>HR: 0.71 (0.59, 0.84)<br>PFS<br>Median: 6.3 (6.2, 7.1)<br>HR: 0.62 (0.52, 0.73)<br>ORR: 45% (40, 51)
Cervical cancer	Multicenter, double-blind; KEYNOTE-A18<br>Cohort 1: Pembro 200 mg Q3W and cisplatin 40 mg/m2 followed by radiation therapy<br>Cohort 2: Placebo and cisplatin 40 mg/m2 followed by radiation therapy	Placebo combination	At prespecified analyses<br>OS<br>Median not reported<br>HR: 0.65 (0.47, 0.90)<br>PFS<br>Median not reached<br>HR: 0.59 (0.43, 0.81)<br>ORR: not reported in PI

Hepatocellular carcinoma	Multicenter, double-blind; KEYNOTE-394<br>Cohort 1: Pembro 200 mg Q3W<br>Cohort 2: Placebo	Placebo	OS at prespecified final analysis<br>Median: 13.9 (12.5, 17.9)<br>HR: 0.78 (0.61, 0.99)<br>PFS at prespecified interim analysis<br>Median: 2 (1.4, 2.7)<br>HR: 0.78 (0.61, 1.00)<br>ORR: 11% (7, 16)
Biliary tract cancer	Multicenter, double-blind; KEYNOTE-966<br>Cohort 1: Pembro 200 mg Q3W, gemcitabine 1000 mg/m2 and cisplatin 25 mg/m2<br>Cohort 2: Placebo, gemcitabine 1000 mg/m2 and cisplatin 25 mg/m2	Placebo combination	OS<br>Median: 12.7 (11.5, 13.6)<br>HR: 0.83 (0.72, 0.95)<br>PFS<br>Median: 6.5 (5.7, 6.9)<br>HR: 0.86 (0.75, 1.00); p-value not significant<br>ORR: 29% (25, 33)
Renal cell carcinoma	Multicenter, open-label; KEYNOTE-426<br>Cohort 1: Pembro 200 mg Q3W and axitinib 5 mg<br>Cohort 2: Sunitinib 50 mg	Sunitinib	OS (updated)<br>Median: 45.7(43.6, NR)<br>HR: 0.73 (0.60, 0.88)<br>PFS<br>Median: 15.1 (12.6, 17.7)<br>HR: 0.69 (0.56, 0.84)<br>ORR: 59% (54, 64)
Endometrial carcinoma	Multicenter, double-blind; KEYNOTE-868/NRG-GY018<br>Cohort 1: Pembro 200 mg Q3W, paclitaxel 175 mg/m2 and carboplatin 5 mg/mL/min<br>Cohort 2: Placebo, paclitaxel 175 mg/m2 and carboplatin 5 mg/mL/min	Placebo combination	dMMR population PFS<br>Median not reached<br>HR: 0.30 (0.19, 0.48)<br>pMMR population PFS<br>Median: 11.1 (8.7, 13.5)<br>HR: 0.60 (0.46, 0.78)<br>OS was not mature at the time of reporting<br>ORR not reported
Triple negative breast cancer	Multicenter, double-blind; KEYNOTE-355<br>Cohort 1: Pembro 200 mg Q3W, paclitaxel, gemcitabine and carboplatin<br>Cohort 2: Placebo, paclitaxel, gemcitabine and carboplatin	Placebo combination	OS at pre-specified final analysis<br>Median: 23 (19.0, 26.3)<br>HR: 0.73 (0.55, 0.95)<br>PFS at pre-specified interim analysis<br>Median: 9.7 (7.6, 11.3)<br>HR: 0.65 (0.49, 0.86)<br>ORR: 53% (46, 59)
Ovarian cancer	Multicenter, double-blind; KEYNOTE-B96<br>Cohort 1: Pembro 200 mg Q3W plus paclitaxel 80 mg/m2 with or without bevacizumab 10 mg/kg<br>Cohort 2: Placebo plus paclitaxel 80 mg/m2 with or without bevacizumab 10 mg/kg	Placebo combination	PFS<br>Median: 8.3 (7.0, 9.4)<br>HR: 0.72 (0.58, 0.89)<br>OS<br>Median: 18.2 (15.3, 21.0)<br>HR: 0.76 (0.61, 0.94)<br>ORR: not reported in the PI

*source, package insert from 02/2026. Please refer to latest PI for accurate information on study details and dose regimen. Only first-line indications or advanced unresectable tumors are listed in the table. Refer to pembrolizumab PI for comprehensive list of indications and efficacy. Refer to respective PIs for clinical outcomes reported for other PD-1 blockers[1]

References

1. Zhou, X.; Yao, Z.; Bai, H.; Duan, J.; Wang, Z.; Wang, X.; Zhang, X.; Xu, J.; Fei, K.; Zhang, Z.; et al. Treatment-Related Adverse Events of PD-1 and PD-L1 Inhibitor-Based Combination Therapies in Clinical Trials: A Systematic Review and Meta-Analysis. *Lancet Oncol* **2021**, *22*, 1265–1274, doi:10.1016/S1470-2045(21)00333-8.
